# Supplementary figures and images for: Machine learning based CRISPR gRNA design for therapeutic exon skipping
Source: PLoS Comput Biol. 2021 Jan 8;17(1):e1008605. doi: 10.1371/journal.pcbi.1008605 (PMC7819613; doi:10.1371/journal.pcbi.1008605)

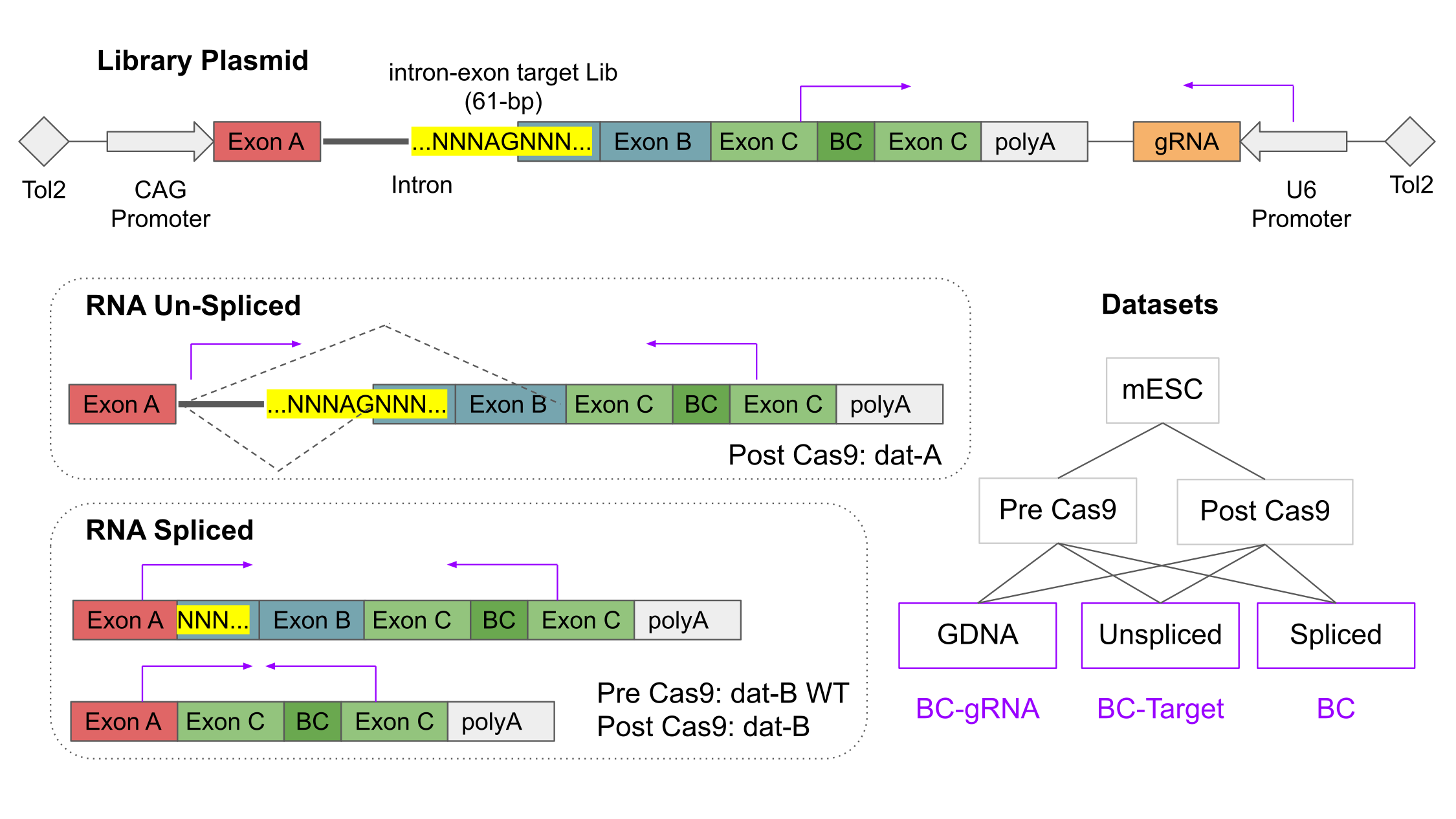

Supplement: S1 Fig — A schematic of the genome-integrating reporter plasmid in which a library of 61-bp human splice-acceptor intron-exon junctions is paired with Cas9 gRNA spacers. A barcode (BC) is embedded in Exon C and captured by sequencing primers (purple arrows) in genomic DNA and RNA transcripts. The deep sequencing of samples from mESCs pre and post Cas9 exposure produces the gDNA, RNA Un-Spliced, and RNA Spliced datasets, which provide barcode to gRNA association, barcode to target sequence association, and just barcode if Exon B is skipped or barcode with the Exon B portion of the target sequence otherwise, respectively. dat-A is constructed from the Post Cas9 RNA Unspliced sequences, which provide information on the genotypic outcomes and frequencies of splice acceptor repair. dat-B is derived from the Post Cas9 RNA Spliced sequences, from which Exon B skipping frequencies are elucidated. Similarly, dat-B WT is derived from Pre Cas9 RNA Spliced sequences. (TIF) [file pcbi.1008605.s001.tif]

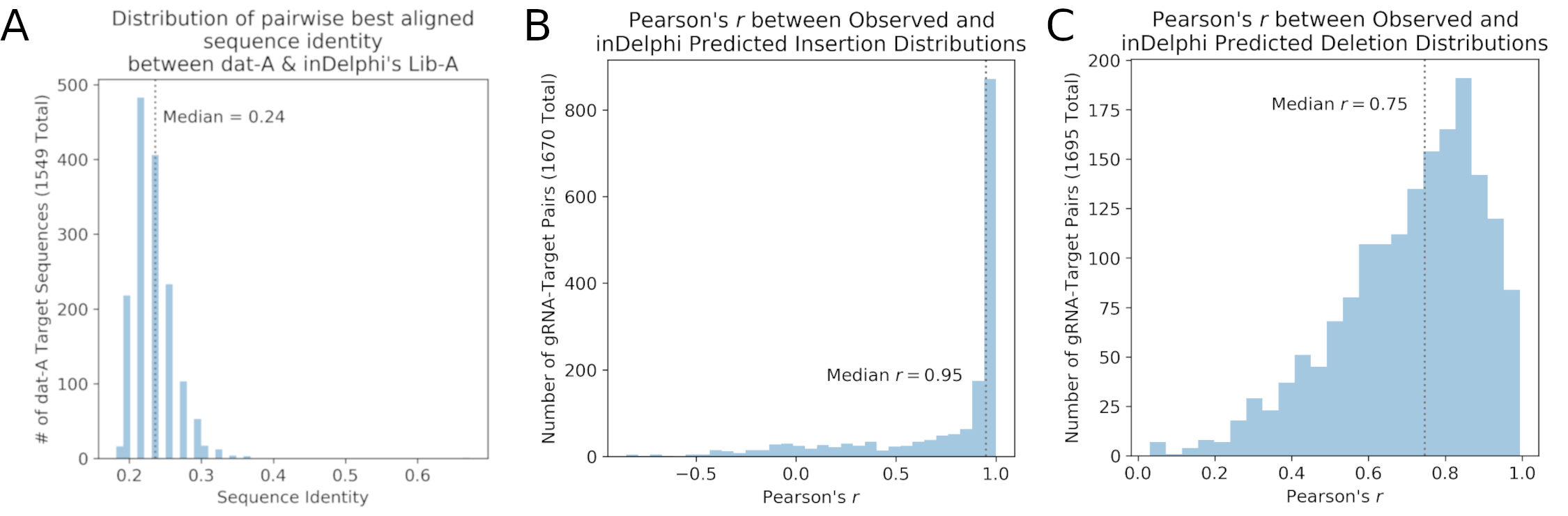

Supplement: S2 Fig — (A) The distribution of sequence identities between most similar pairs of sequences (as determined through local sequence alignment) from dat-A, and the set of sequences inDelphi was trained on (median sequence identity is 0.24). (B) The distribution of Pearson’s r between observed and inDelphi predicted 1-bp insertion (median r = 0.95). (C) The distribution of Pearson’s r between observed and inDelphi predicted deletions (median r = 0.75). (TIF) [file pcbi.1008605.s002.tif]

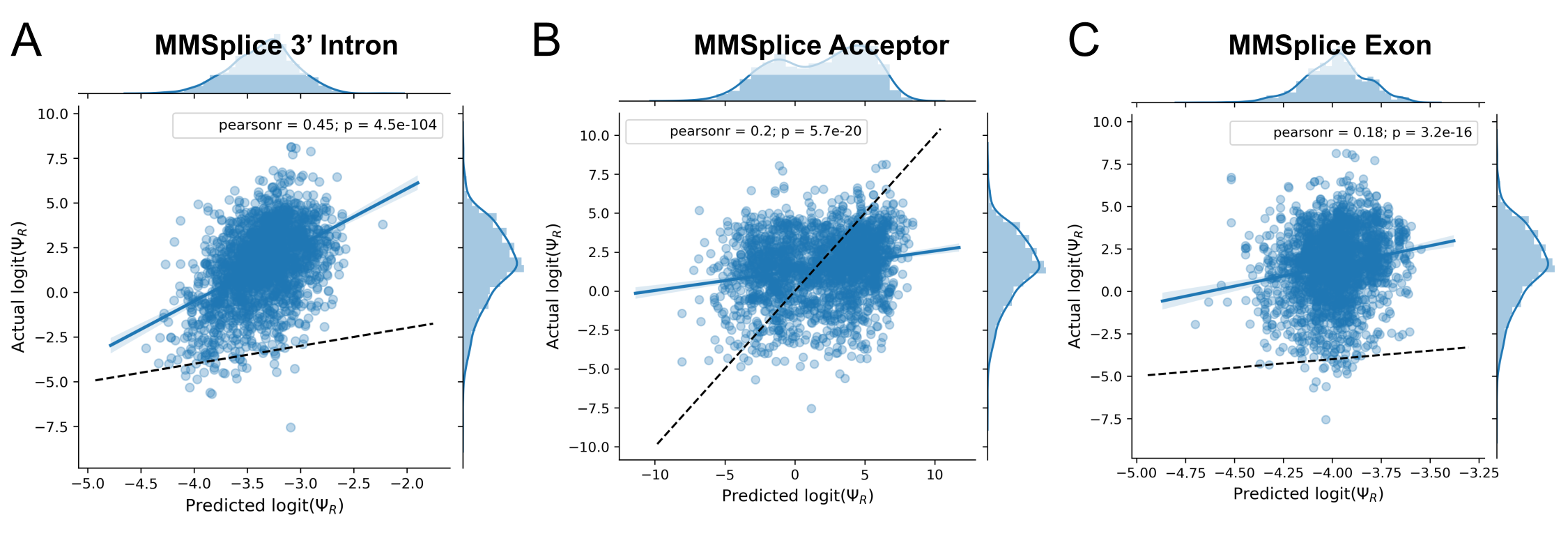

Supplement: S3 Fig — In all the plots, the black dashed line represents the identity line where actual equals predicted. (A) Actual logit(ΨR) vs MMSplice intron module predicted logit(ΨR) (r = 0.45). (B) Actual logit(ΨR) vs MMSplice acceptor module predicted logit(ΨR) (r = 0.20). (C) Actual logit(ΨR) vs MMSplice exon module predicted logit(ΨR) (r = 0.18). (TIF) [file pcbi.1008605.s003.tif]

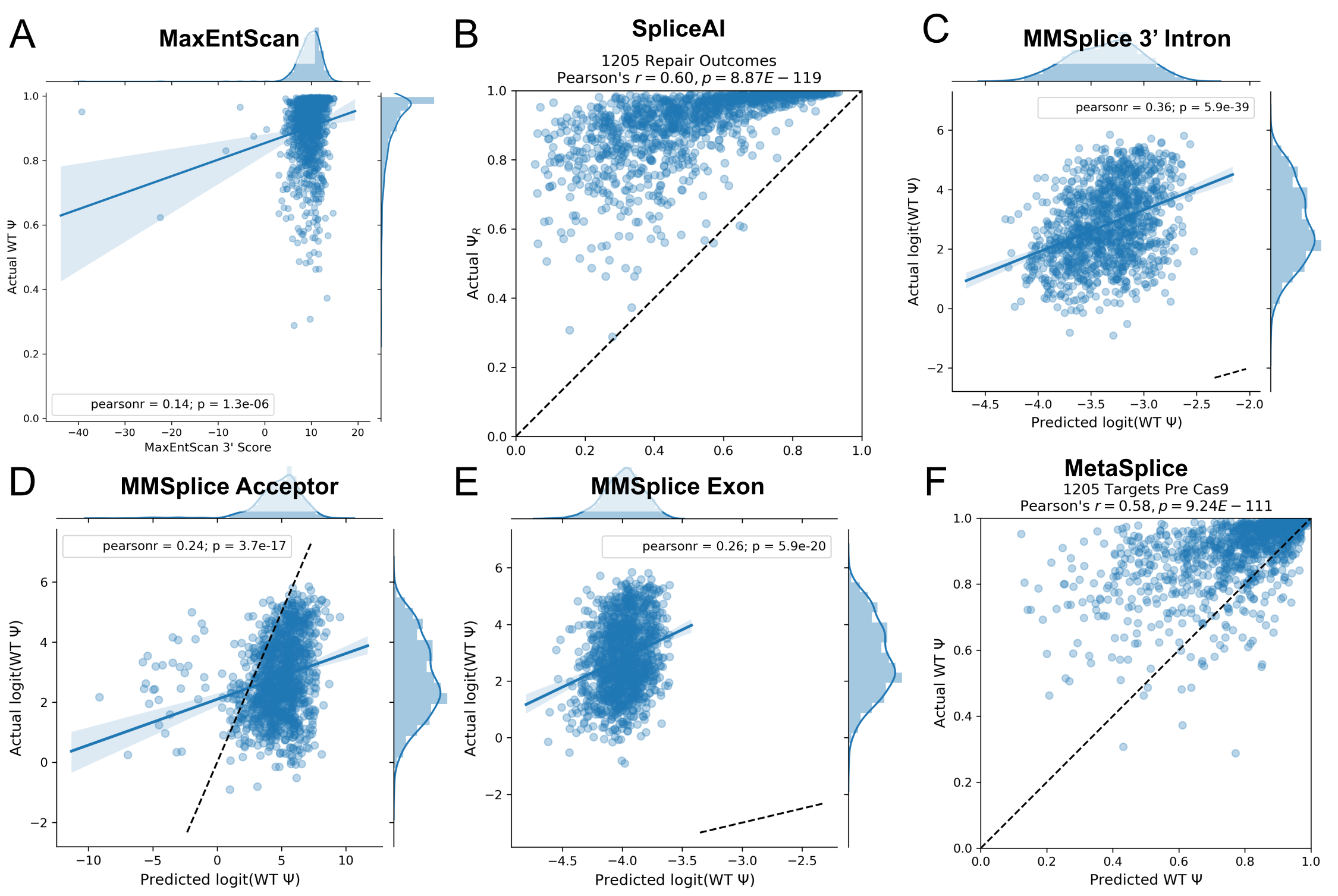

Supplement: S4 Fig — dat-B WT originally represents 1,697 lib-SA targets, but for this analysis, only the 1,205 with at least 50 sequencing reads for estimating Ψ were considered. In all the plots, the black dashed line represents the identity line where actual equals predicted. (A) Actual WT Ψ vs. MaxEntScan 3’ Score of the acceptor sequence (r = 0.14). (B) Actual vs. SpliceAI predicted WT Ψ (r = 0.6). (C) Actual logit(ΨR) vs MMSplice intron module predicted logit(ΨR) (r = 0.36). (D) Actual logit(ΨR) vs MMSplice acceptor module predicted logit(ΨR) (r = 0.24). (E) Actual logit(ΨR) vs MMSplice exon module predicted logit(ΨR) (r = 0.26). (F) Actual vs MetaSplice predicted WT ΨR (r = 0.58), where MetaSplice is tuned using the entire dat-B dataset. (TIF) [file pcbi.1008605.s004.tif]

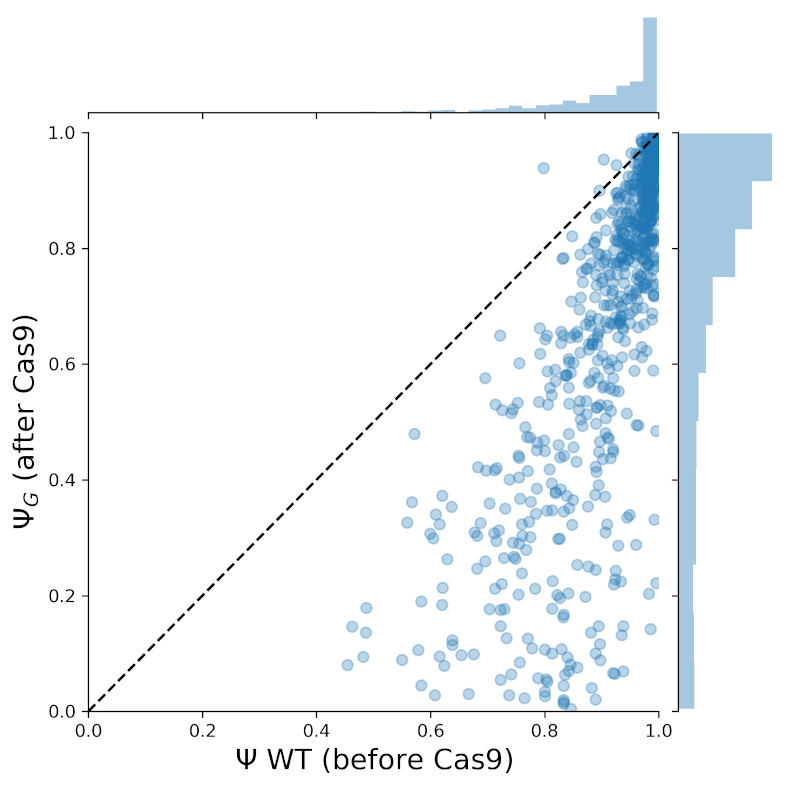

Supplement: S5 Fig — Plot of ΨG vs WT Ψ of 735 gRNAs and their corresponding lib-SA targets. After Cas9-mediated repair, all but 23 exhibited a lower ΨG compared to that of WT Ψ (points below diagonal line). Those 23 showed only a small mean increase in PSI of 0.01. The mean WT Ψ is 0.91, and shifted lower to a mean ΨG of 0.68 after Cas-9 treatment. (TIF) [file pcbi.1008605.s005.tif]

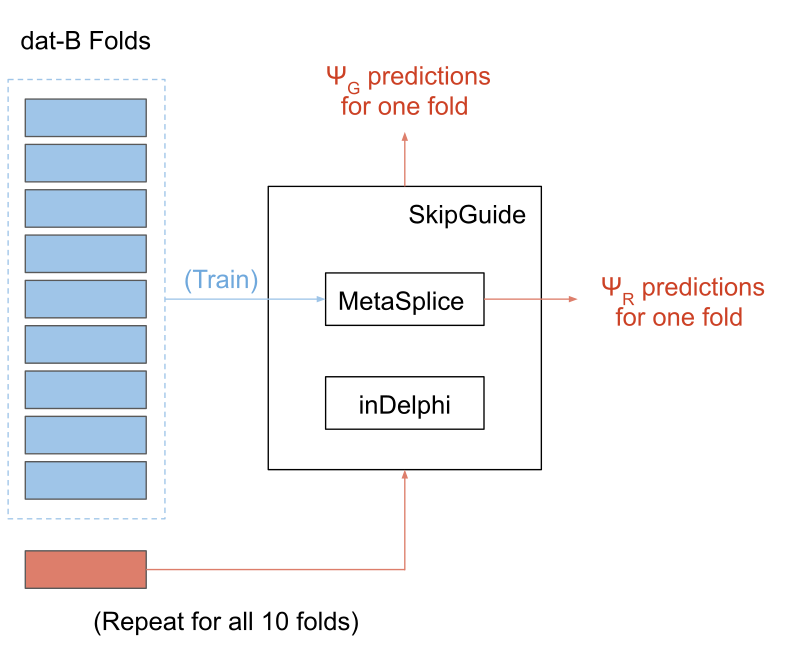

Supplement: S6 Fig — The same 10 folds used to evaluate MetaSplice were used to evaluate SkipGuide: the 2,113 repair outcomes in dat-B were first grouped into the 1,063 associated gRNAs, and then randomly partitioned into 10 folds. This ensures that no two folds contain repair outcomes from the same associated gRNA. Predictions for gRNAs in one fold are obtained by using the other 9 folds to fit MetaSplice’s linear model weights within SkipGuide. We repeated this for all 10 folds to obtain ΨR predictions for each of the 2,113 repair outcomes, and ΨG predictions for each of the 1,063 gRNAs. (TIF) [file pcbi.1008605.s006.tif]

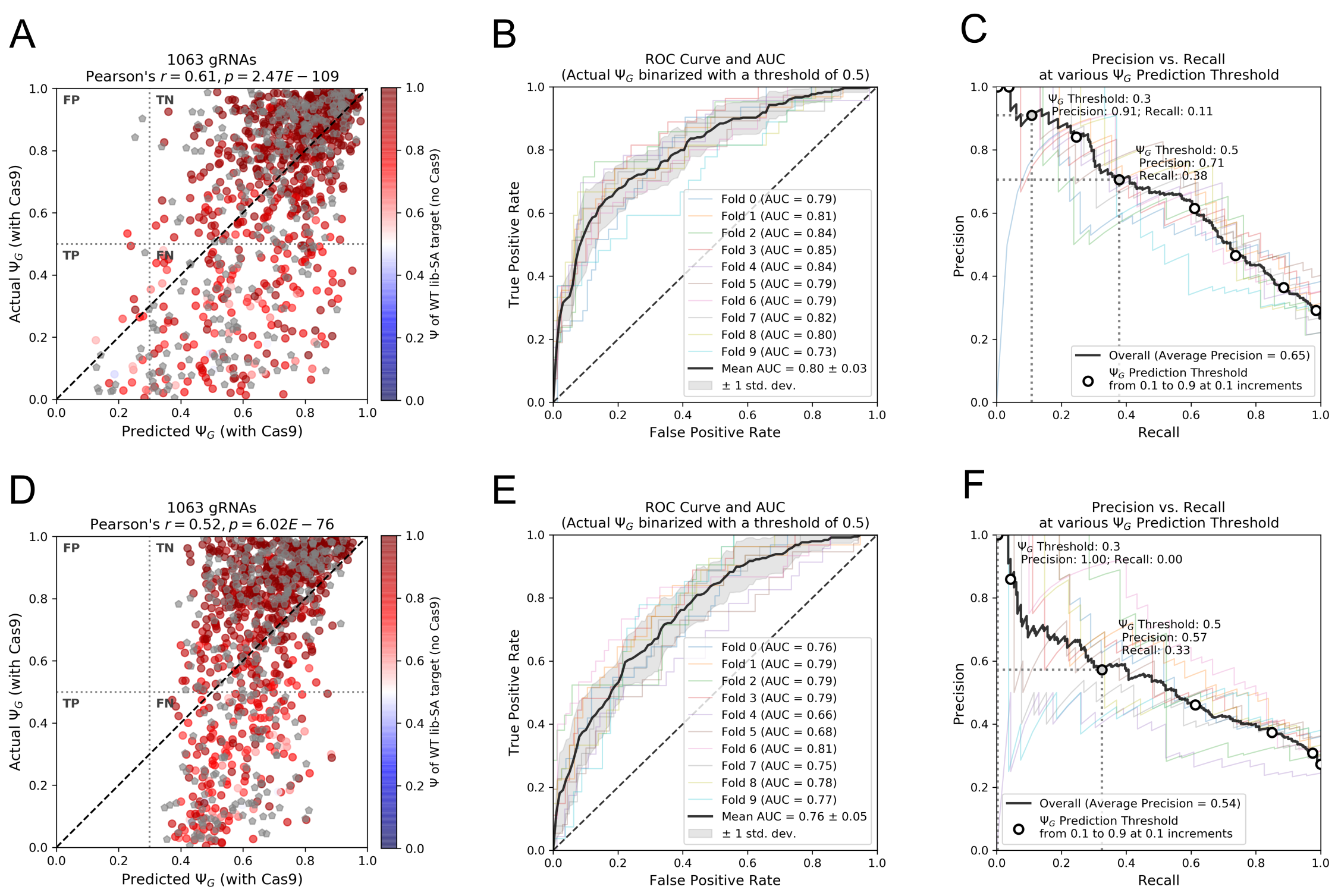

Supplement: S7 Fig — (A), (B), (C) The 10-fold cross validation performance of SkipGuide when wMMSplice is used instead of MetaSplice. Mean r = 0.61, mean MAE = 0.17, mean MSE = 0.05, mean RMSE = 0.23, and mean AUC = 0.81, over the 10 folds. (D), (E), (F) The performance of SkipGuide when a linear model over SpliceAI prediction (similar to that of wMMSplice and MetaSplice) is used instead of MetaSplice. Mean r = 0.52, mean MAE = 0.20, mean MSE = 0.06, mean RMSE = 0.25, and mean AUC = 0.76, over the 10 folds. (TIF) [file pcbi.1008605.s007.tif]

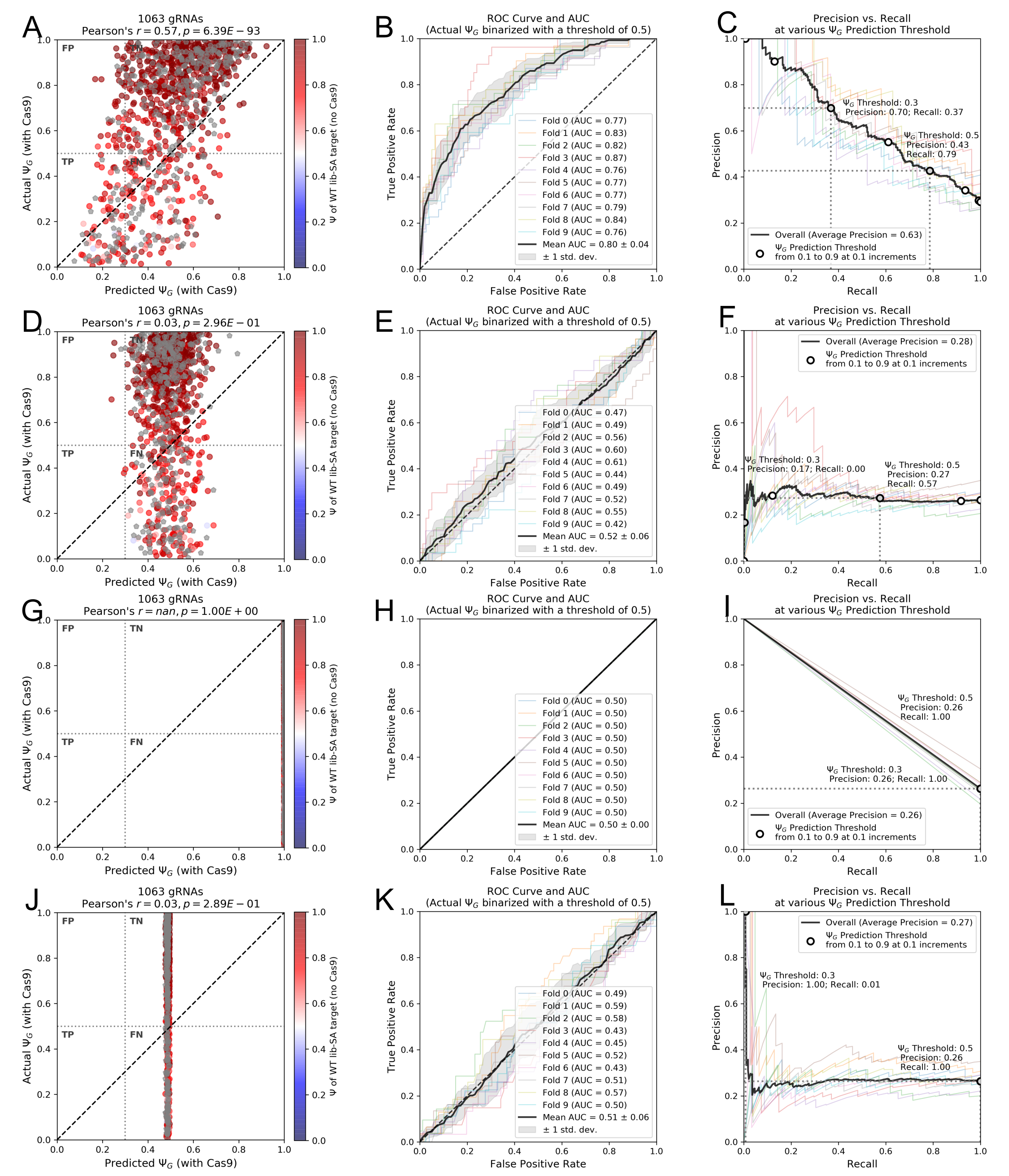

Supplement: S8 Fig — The results of a perturbation study performed on SkipGuide, where we perform the same performance evaluations depicted in Fig 4, wherein one of, or both of, the SkipGuide prediction modules are replaced with a dysfunctional predictor. (A), (B), (C) inDelphi is replaced with a predictor that always outputs uniform frequencies for all possible repair genotypes. (D), (E), (F) MetaSplice is replaced with a predictor that outputs random values between 0 and 1. (G), (H), (I) MetaSplice is replaced with a predictor that always outputs 1. Note that (I) should not be interpreted, as the precision is not actually defined at recall less than 1 (because TP = 0 and FP = 0, so precision is indeterminate). (D) Both the inDelphi perturbation that produced (A), (B), (C) and the MetaSplice perturbation that produced (D), (E), (F) were performed. Note that the precision = 1 at 0.3 threshold in (F) should not be interpreted, as precision is actually undefined at the 0.3 threshold (TP = 0 and FP = 0). (TIF) [file pcbi.1008605.s008.tif]

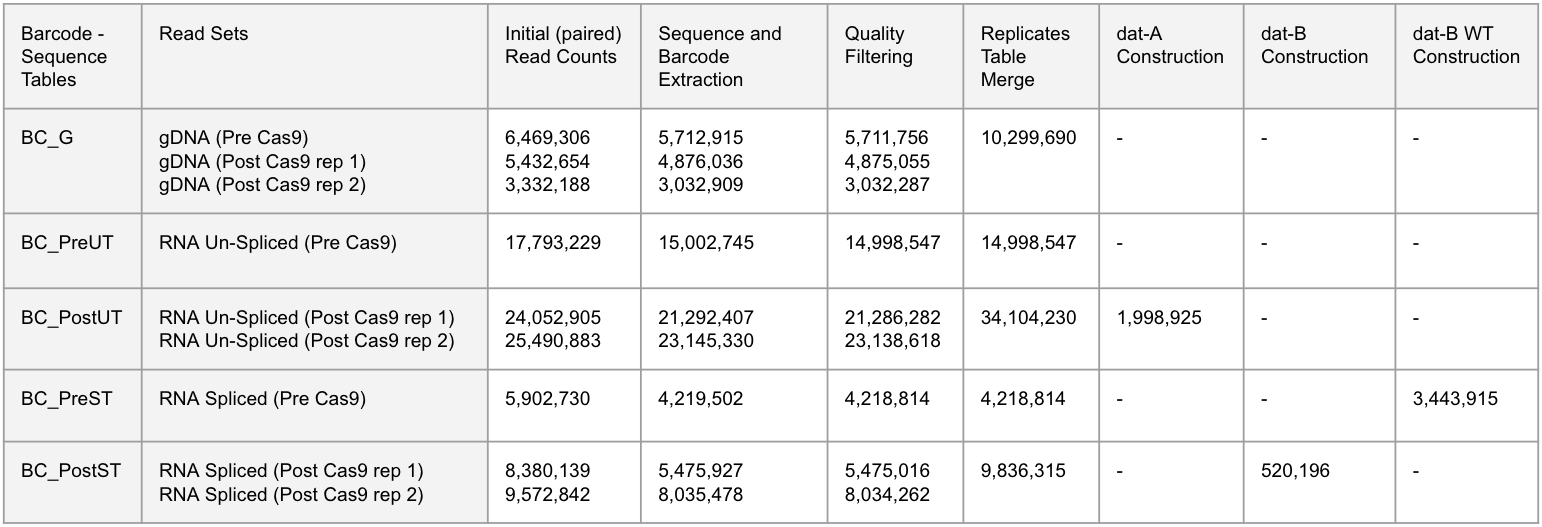

Supplement: S9 Fig — The sequence read counts retained at each stage of processing as described in the Methods. (TIF) [file pcbi.1008605.s009.tif]

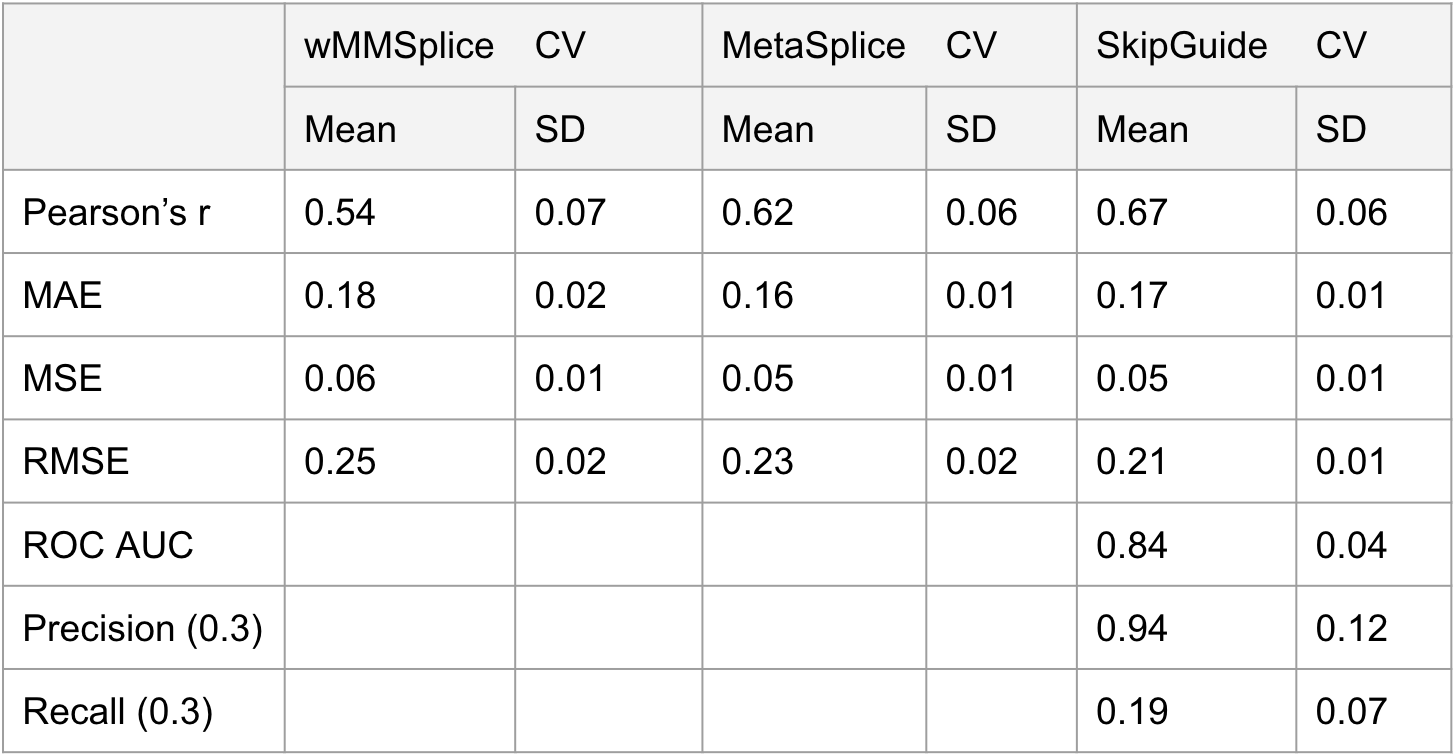

Supplement: S10 Fig — Depending on the data split that produces 10 folds, the evaluated performance may vary. To assess robustness, 1,000 repeats of the cross validation with random 10 fold splits were performed. This procedure would result in 10,000 fold predictions, which would provide 10,000 metric values for a given metric. The metric values shown are averages over the 10,000 metric values. SD denotes standard deviation, and precision and recall were calculated using a prediction threshold of 0.3. (TIF) [file pcbi.1008605.s010.tif]
