# Supplementary material for: Machine learning based CRISPR gRNA design for therapeutic exon skipping
Source: PLoS Comput Biol. 2021 Jan 8;17(1):e1008605. doi: 10.1371/journal.pcbi.1008605 (PMC7819613; doi:10.1371/journal.pcbi.1008605)
Supplement: S3 Text — (DOCX) [file pcbi.1008605.s013.docx]

SpCas9 gRNA/splice-acceptor library design

CGCGTGTCTATATGTGTAATCANNNNNNNNNNNNNNNNNNNNNNNNCTNNNNNNNNNNNNNNNNNNNNNNNNNNNNNNNNNNNGTCGACgagggcagctggtAATATTGTGGAAAGGACGAAACACCGNNNNNNNNNNNNNNNNNNNNGTTTAAGAGCTATGCTGGAAAC

Splice-acceptor/SpCas9 gRNA in plasmid backbone

caagatccgccacaacatcgaggtaagttatcaccttcgtggctacagagtttccttatttgtctctgttgccggcttatatggacaagcatatcacagccatttatcggagcgcctccgtacacgctattatcggacgcctcgcgagatcaatacgattaccagctgccctcgTCGaCNNNNNNNNNNNNNNNNNNNNNNNNNNNNNNNNNNNAGNNNNNNNNNNNNNNNNNNNNNNNNtgattacacatatagacacgcGAGCAGCCATCTTTTATAGAATGGGtagaacccgtcctaaggactcagattgagcatcgtttgcttctcgagtactacctggtacagatgtctcttcaaacaggacggcagcgtgcagctcgccNNNNNNNNNNNNNNNgaccactaccagcagaacacccccatcggcgacggccccgtgctgctgcccgacaaccactacctgagctaccagtccgccctgagcaaagaccccaacgagaagcgcgatcacatggtcctgctggagttcgtgaccgccgccgggatcactctcggcatggacgagctgtacaaggactgagactgatagtaaggcccattacctgcACCGGTNNNNNNNNNNNNNNNNNNACGCGTgcagaacacagcggttcgactgtgccttctagttgccagccatctgttgtttgcccctcccccgtgccttccttgaccctggaaggtgccactcccactgtcctttcctaataaaatgaggaaattgcatcgcattgtctgagtaggtgtcattctattctggggggtggggtggggcaggacagcaagggggaggattgggaagacaatagcaggcatgctggggatgcggtgggctctatggGTTAACTTCTAGCTCTAAAACAAAAAAGCACCGACTCGGTGCCACTTTTTcaagttgataacggacTAGCCTTATTTAAACTTGCTATGCTgtttccagcatagctcttaaacNNNNNNNNNNNNNNNNNNNNCGGTGTTTCGTCCTTTCCACAAGATATATAAAGCCAAGAAATCGAAATACTTTCAAGTTACGGTAAGCATATGATAGTCCATTTTAAAACATAATTTTAAAACTGCAAACTACCCAAGAAATTATTACTTTCTACGTCACGTATTTTGTACTAATATCTTTGTGTTTACAGTCAAATTAATTCCAATTATCTCTCTAACAGCCTTGTATCGTATATGCAaatatgaaggaatcatgggaaataggccctCAATTG

1. **Amplify splicing oligo library in 3 PCR steps, adding post-splice acceptor sequence to 5’ end and gRNA hairpin sequence to 3’ of the oligo**

PCRA1 **Ta = 60**

110518_SALib_PCRA1_fw gagtccttaggacgggttctaCCCATTCTATAAAAGATGGCTGCTCgcgtgtctatatgtgtaatca

030918_SALib_Lib1_GArv GTTGATAACGGACTAGCCTTATTTAAACTTGCTATGCTGTTTCCAGCATAGCTCTTAAAC

**PCRA1**

xx ul 2% of 10 ng/uL Oligo library (>5 ng)

25 ul       2x Q5 UltraII Mastermix

1.25 ul        20uM Fw primer: 110518_SALib_PCRA1_fw

1.25 ul        20uM Rv primer: 030918_SALib_Lib1_GArv

xx ul    mQ (to final volume 50ul)

98°C   |  98°C    60°C    72°C  | 72°C |    4**°**C => 8 cycles

30sec | 10sec 30sec **1min** | 5min |  forever

PCR purify product in 50 uL EB

Use 22.5 uL as input to PCRA2

**PCRA2**

110518_SALib_PCRA2_fw gctgccgtcctgtttgaagagacatctgtaccaggtagtactcgagaagcaaacgatgctcaatctgagtccttaggacgggttctaCCC

110518_SALib_PCRA23_rv GTTGATAACGGACTAGCCTTATTTAAACTTGCTATGCTG

22.5 ul PCRA1 product

25 ul       2x Q5 UltraII Mastermix

1.25 ul        20uM Fw primer: 110518_SALib_PCRA2_fw

1.25 ul        20uM Rv primer: 110518_SALib_PCRA23_rv

xx ul    mQ (to final volume 50ul)

98°C   |  98°C       72°C  | 72°C |    4**°**C => 7 cycles

30sec | 10sec **1min** | 5min |  forever

PCR purify product

**PCRA3**

110518_SALib_PCRA3_fw cggggccgtcgccgatgggggtgttctgctggtagtggtcNNNNNNNNNNNNNNNggcgagctgcacgctgccgtcctgtttgaagagac

110518_SALib_PCRA23_rv GTTGATAACGGACTAGCCTTATTTAAACTTGCTATGCTG

22.5 ul PCRA2 product

50 ul       2x Q5 UltraII Mastermix

2.5 ul        20uM Fw primer: 110518_SALib_PCRA3_fw

2.5 ul        20uM Rv primer: 110518_SALib_PCRA23_rv

22.5 ul    mQ (to final volume 50ul)

98°C   |  98°C       72°C  | 72°C |    4**°**C => 7 cycles (as determined by qPCR)

30sec | 10sec **1min** | 5min |  forever

**Gel extraction**

Isolate 406-bp band from 2% SYBR gel with Qiagen gel cleanup.

**Amplified sequence**

gtggttgtcgggcagcagcacggggccgtcgccgatgggggtgttctgctggtagtggtcNNNNNNNNNNNNNNNggcgagctgcacgctgccgtcctgtttgaagagacatctgtaccaggtagtactcgagaagcaaacgatgctcaatctgagtccttaggacgggttctaCCCATTCTATAAAAGATGGCTGCTCgcgtgtctatatgtgtaatcaNNNNNNNNNNNNNNNNNNNNNNNNCTNNNNNNNNNNNNNNNNNNNNNNNNNNNNNNNNNNNGTCGACgagggcagctggtAATATTGTGGAAAGGACGAAACACCGNNNNNNNNNNNNNNNNNNNNGTTTAAGAGCTATGCTGGAAACAGCATAGCAAGTTTAAATAAGGCTAGTCCGTTATCAAC

1. **Amplify diverse barcoded fragment from p2T CAG SpliceAcc-Barcoded U6 2x BbsI HygR**

PCRB1 **Ta = 66 598bp**

110518_SALib_PCRB1_GAfw GACCACTACCAGCAGAACACC

030918_SALib_Plas1_GArv gtttaagagctatgctggaaacAGCA

**Amplified sequence**

gaccactaccagcagaacacccccatcggcgacggccccgtgctgctgcccgacaaccactacctgagctaccagtccgccctgagcaaagaccccaacgagaagcgcgatcacatggtcctgctggagttcgtgaccgccgccgggatcactctcggcatggacgagctgtacaaggactgagactgatagtaaggcccattacctgcACCGGTNNNNNNNNNNNNNNNNNNNNACGCGTgcagaacacagcggttcgactgtgccttctagttgccagccatctgttgtttgcccctcccccgtgccttccttgaccctggaaggtgccactcccactgtcctttcctaataaaatgaggaaattgcatcgcattgtctgagtaggtgtcattctattctggggggtggggtggggcaggacagcaagggggaggattgggaagacaatagcaggcatgctggggatgcggtgggctctatggGTTAACTTCTAGCTCTAAAACAAAAAAGCACCGACTCGGTGCCACTTTTTcaagttgataacggacTAGCCTTATTTAAACTTGCTATGCTgtttccagcatagctcttaaac

**QPCR to determine cycle number for amplification PCR**

0.2 ul p2T CAG SpliceAcc-Barcoded U6 2x BbsI HygR

7.5 ul       2x Q5 UltraII Mastermix

0.375ul        20uM Fw primer: 110518_SALib_PCRB1_GAfw

0.375ul        20uM Rv primer: 030918_SALib_Plas1_GArv

0.75 ul 20X EVA Green

5.8 ul    mQ (to final volume 20ul)

98°C   |  98°C    66°C    72°C  | 72°C |    4**°**C

30sec | 10sec 30sec 60sec | 5min |  forever    => 40 cycles

- Run product on gel to visualize 598bp. *Note: size may appear larger due to dye.*
- Determine top of S-curve cycle count. Reduce to scale based on input real PCR vs QPCR.

**Diverse plasmid PCR**

- xx ul 1 ug p2T CAG SpliceAcc-Barcoded U6 2x BbsI HygR
- 50 ul       2x Q5 UltraII Mastermix
- 2.5 ul        20uM Fw primer: 110518_SALib_PCRB1_GAfw
- 2.5 ul        20uM Rv primer: 030918_SALib_Plas1_GArv
- xx ul    mQ (to final volume 50ul)
- 98°C   |  98°C    66°C    72°C  | 72°C |    4**°**C => xx cycles determined by QPCR
- 30sec | 10sec 30sec **1min** | 5min |  forever
- **Gel extraction**
- Isolate 598bp band from 2% SYBR gel with Qiagen gel cleanup.

1. **Gibson Assembly (backbone:insert is molar 2:1 to reduce recombination)**

xx ul 789 ng Donor plasmid fragment (PCRB1 product, 598 bp)

xx ul 267 ng Insert template oligo library (PCRA3 product, 406 bp)

30ul 2x GA MM

xx ul    mQ (to final volume 60ul)                  => 1h @ 50C, then 4 deg

Can scale down input to account for lower purified product amounts, ensuring the ratio is maintained.

1. **Remove unligated fragments**

Add to GA mixture

9ul 10x Plasmid Safe Buffer (Epicenter)

9ul ATP (25mM)

3ul Plasmid Safe Nuclease

9ul mQ (to final volume 90ul)                  => 1h @ 37C, optional 70 deg for 30 min then 4 deg or

proceed straight to step 5

1. **Cleanup and linearization**

PCR purify and elute in 50ul mQ

**Digest**

50ul PCR purified circularized library

10ul 10x CutSmart Buffer

3ul SspI

37ul mQ (to final volume 100ul)                   => 3h to o/n @ 37C

**PCR purification**

Perform Qiagen PCR purification, eluting in 40ul

**Linearized product 884bp**

ATTaccagctgccctcGTCGACNNNNNNNNNNNNNNNNNNNNNNNNNNNNNNNNNNNAGNNNNNNNNNNNNNNNNNNNNNNNNTGATTACACATATAGACACGCGAGCAGCCATCTTTTATAGAATGGGtagaacccgtcctaaggactcagattgagcatcgtttgcttctcgagtactacctggtacagatgtctcttcaaacaggacggcagcgtgcagctcgccNNNNNNNNNNNNNNNgaccactaccagcagaacacccccatcggcgacggccccgtgctgctgcccgacaaccactacctgagctaccagtccgccctgagcaaagaccccaacgagaagcgcgatcacatggtcctgctggagttcgtgaccgccgccgggatcactctcggcatggacgagctgtacaaggactgagactgatagtaaggcccattacctgcACCGGTNNNNNNNNNNNNNNNNNNNNACGCGTgcagaacacagcggttcgactgtgccttctagttgccagccatctgttgtttgcccctcccccgtgccttccttgaccctggaaggtgccactcccactgtcctttcctaataaaatgaggaaattgcatcgcattgtctgagtaggtgtcattctattctggggggtggggtggggcaggacagcaagggggaggattgggaagacaatagcaggcatgctggggatgcggtgggctctatggGTTAACTTCTAGCTCTAAAACAAAAAAGCACCGACTCGGTGCCACTTTTTcaagttgataacggacTAGCCTTATTTAAACTTGCTATGCTgtttccagcatagctcttaaacNNNNNNNNNNNNNNNNNNNNCGGTGTTTCGTCCTTTCCACAAT

1. **p2T CAG SpliceAcc-Barcoded U6 2x BbsI HygR backbone preparation**

**Digest**

6 ug p2T CAG SpliceAcc-Barcoded U6 2x BbsI HygR

10 ul 10x 2.1 Buffer

3 ul BbsI

2 ul SalI-HF

XX ul mQ dH2O (to final volume 100ul)                   => 3h to o/n @ 37C

**Gel extraction**

Isolate 8,105 bp band (expect to see 746 bp band as well) from 1% SYBR gel, elute in 30ul with Qiagen gel purification kit.

1. **Amplify SspI-linearized splicing library and add intron overhang at 5’ and U6-homology overhang at 3’ for GA-cloning**

PCRC1 **Ta = 67** **959 bp**
030918_SALib_Lib2_GAfw gtacacgctattatcggacgcctcgcgagatcaatacgattaccagctgccctcgtcgaC

010415_sgRNA_60bp_fw TAACTTGAAAGTATTTCGATTTCTTGGCTTTATATATCTTGTGGAAAGGACGAAACACCG

**QPCR to determine cycle number for amplification PCR**

0.2 uL SspI-digested, PCR-purified product

7.5 ul       2x Q5 UltraII Mastermix

0.375 ul        20uM Fw primer: 030918_SALib_Lib2_GAfw

0.375 ul        20uM Rv primer: 010415_sgRNA_60bp_fw

0.75 ul 20X Eva Green

5.8 ul    mQ dH2O (to final volume 15 ul)

98°C   |  98°C    67°C    72°C  | 72°C |    4**°**C

30sec | 10sec 30sec 60sec | 5min |  forever    => 40 cycles

- Run product on gel to visualize 944 bp band.
- For PCR, use the number of cycles in the qPCR Ct value.

**PCRC1 product 959 bp**

gtacacgctattatcggacgcctcgcgagatcaatacgATTaccagctgccctcGTCGACNNNNNNNNNNNNNNNNNNNNNNNNNNNNNNNNNNNAGNNNNNNNNNNNNNNNNNNNNNNNNTGATTACACATATAGACACGCGAGCAGCCATCTTTTATAGAATGGGtagaacccgtcctaaggactcagattgagcatcgtttgcttctcgagtactacctggtacagatgtctcttcaaacaggacggcagcgtgcagctcgccNNNNNNNNNNNNNNNgaccactaccagcagaacacccccatcggcgacggccccgtgctgctgcccgacaaccactacctgagctaccagtccgccctgagcaaagaccccaacgagaagcgcgatcacatggtcctgctggagttcgtgaccgccgccgggatcactctcggcatggacgagctgtacaaggactgagactgatagtaaggcccattacctgcACCGGTNNNNNNNNNNNNNNNNNNNNACGCGTgcagaacacagcggttcgactgtgccttctagttgccagccatctgttgtttgcccctcccccgtgccttccttgaccctggaaggtgccactcccactgtcctttcctaataaaatgaggaaattgcatcgcattgtctgagtaggtgtcattctattctggggggtggggtggggcaggacagcaagggggaggattgggaagacaatagcaggcatgctggggatgcggtgggctctatggGTTAACTTCTAGCTCTAAAACAAAAAAGCACCGACTCGGTGCCACTTTTTcaagttgataacggacTAGCCTTATTTAAACTTGCTATGCTgtttccagcatagctcttaaacNNNNNNNNNNNNNNNNNNNNCGGTGTTTCGTCCTTTCCACAAGATATATAAAGCCAAGAAATCGAAATACTTTCAAGTTA

**Library PCR**

20 ul 50% of linearized splicing oligo product

50 ul       2x Q5 UltraII Mastermix

2.5 ul        20uM Fw primer: 030918_SALib_Lib2_GAfw

2.5 ul        20uM Rv primer: 010415_sgRNA_60bp_fw

25 ul    mQ dH2O (to final volume 100ul)

98°C   |  98°C    67°C    72°C  | 72°C |    4**°**C => xx cycles determined by QPCR

30sec | 10sec 30sec **1min** | 5min |  forever

**Gel extraction**

Isolate 944 bp band from 2% SYBR gel with Qiagen gel cleanup. Elute in 30 uL EB.

1. **Gibson Assembly (backbone:insert is molar 1:3, ng amounts can be scaled down)**

xx ul 1,970 ng SalI-HF/BbsI-digested p2T CAG SpliceAcc-Barcoded U6 2x BbsI HygR (8,105 bp)

xx ul 714 ng PCRC1 product (959 bp)

30ul 2x GA MM

xx ul    mQ (to final volume 60ul)                  => 1h @ 50C

*-Scale down DNA ng amounts equally and accordingly if there is insufficient of either product to fit into reaction volume*

*-Separately perform a 5 uL scaled down Gibson reaction with 164 ng digested plasmid and no insert as a control.*

1. **Cleanup**

40 ul Gibson Reaction *(store remainder 20ul Gibson reaction at -20C as backup)*

0.4ul GlycoBlue

0.8ul 50mM NaCl

38.8ul Isopropanol

- Vortex, incubate at room temperature for 15min
- SPIN >15.000g for 15min
- Carefully remove liquid without disturbing pellet
- Wash with 300ul 80% EtOH and SPIN >15.000g for 5min
- Carefully remove all liquid with p200 and airdry pellet 1-3min
- Resuspend in 10ul TE/EB/mQ at 55C 10min

1. **Transformation**

Transform 0.25 uL of experimental Gibson and control Gibson and count colonies on 1:10, 1:1,000, 1:100,000 dilution series plates:

*Hope for 100-1000X as many colonies as there are unique library members in experimental Gibson and >10-fold fewer colonies in control Gibson.*

Pick 16 clones to grow up for miniprep or colony PCR (Onetaq Ta=55) with primers below

(Optional) Test digest with XhoI, expect 8.0 kb + 841 bp bands

Send each miniprep/colony PCR for two sequencing reactions with:
1. 051018_Splice_RNA_fw caagatccgccacaacatcg

2. pX330_seq_fw GAGGGCCTATTTCCCATGAT

If confirmed, transform 8 x 1 uL of the Gibson product into 8 x 25 ul NEB10beta electrocompetent cells (100ul electrocompetent NEB10beta per vial) using the protocol at <https://www.neb.com/protocols/0001/01/01/electroporation-protocol-c3020>

After 1 hour of outgrowth as recommended in protocol, pool the entire 8 mL competent cell/media mixture. Plate 1:1,000 and 1:100,000 for counting. Transfer the remaining into 400 mL LB + Amp. Shake overnight, spin down for maxiprep as 2 x 200 mL aliquots, perform Qiagen plasmid maxi on 1 x 200 mL aliquot, keeping the second as backup.

1. **Validation of p2T-sgRNA+Target library/library prep**

**- > (optional): move on to NextSeq for verification**

**To perform library prep on plasmid library, genomic DNA with library pre- or post-Cas9 treatment, or cDNA to isolate unspliced products:**

**PCR1**

051018_SpliceAssay_gDNA_r1seq CTTTCCCTACACGACGCTCTTCCGATCT NNN taccagctgccctcgTCGaC

051018_SpliceAssay_gDNA_r1seq_2N CTTTCCCTACACGACGCTCTTCCGATCT NN taccagctgccctcgTCGaC

051018_SpliceAssay_gDNA_r1seq_1N CTTTCCCTACACGACGCTCTTCCGATCT N taccagctgccctcgTCGaC

051018_SpliceAssay_gDNA_r1seq_0N CTTTCCCTACACGACGCTCTTCCGATCT taccagctgccctcgTCGaC

110518_SpliceAssay_DNARNA_r2seq GGAGTTCAGACGTGTGCTCTTCCGATCT NNN ggggtgttctgctggtagtggtc

110518_SpliceAssay_DNARNA_r2seq_2N GGAGTTCAGACGTGTGCTCTTCCGATCT NN ggggtgttctgctggtagtggtc

110518_SpliceAssay_DNARNA_r2seq_1N GGAGTTCAGACGTGTGCTCTTCCGATCT N ggggtgttctgctggtagtggtc

110518_SpliceAssay_DNARNA_r2seq_0N GGAGTTCAGACGTGTGCTCTTCCGATCT ggggtgttctgctggtagtggtc

**NEBNext Mix**

xxul **1ug** plasmid DNA or **16** ug gDNA in **800 uL** PCR volume

50/400 ul       2x Q5 UltraII Mastermix

2.5/20 ul        20uM equimolar pool of 051018_SpliceAssay_gDNA_r1seq_0N-3N

2.5/20 ul        20uM equimolar pool of 110518_SpliceAssay_DNARNA_r2seq_ON-3N

xxul    mQ dH2O (to final volume **100/800 ul**)

98°C   |  98°C    72°C  | 72°C |    4**°** C => 13 cycles

30sec  | 10sec **1 min** | 5min |  forever

**PCR purify and elute in 50ul.**

**gDNA PCR1 product (326 bp)**

CTTTCCCTACACGACGCTCTTCCGATCTNNNtaccagctgccctcgTCGaCNNNNNNNNNNNNNNNNNNNNNNNNNNNNNNNNNNNAGNNNNNNNNNNNNNNNNNNNNNNNNtgattacacatatagacacgcGAGCAGCCATCTTTTATAGAATGGGtagaacccgtcctaaggactcagattgagcatcgtttgcttctcgagtactacctggtacagatgtctcttcaaacaggacggcagcgtgcagctcgccNNNNNNNNNNNNNNNgaccactaccagcagaacaccccNNNAGATCGGAAGAGCACACGTCTGAACTCC

qPCR **TM 72 406 bp**

**QPCR to determine cycle number for PCR2:**

0.2 ul purified PCR1

7.5 ul       2x Q5 UltraII Mastermix

0.375 ul        20uM Fw primer: 061813_PE1

0.375 ul        20uM Rv primer: 062118_MultiplexRd2_full_noidx

0.75 ul 20X EvaGreen

5.8 ul    mQ (to final volume 15 ul)

98°C |  98°C   72°C  72°C  | 72°C |    4**°**C

30sec | 10sec 30sec 30sec | 5min |  forever    => 40 cycles

These primers are unindexed versions of the primers to be used in PCR2 in order to save money and not use the valuable primers from the indexed kit:

061813_PE1 AATGATACGGCGACCACCGAGATCTACACTCTTTCCCTACACGACGCTCTTCCGATCT

062118_MultiplexRd2_full_noidx CAAGCAGAAGACGGCATACGAGAT GTGACTGGAGTTCAGACGTGTGCTCTTCCGATCT

- Run product on gel to visualize 406 bp band. Not much background?
- Cycle count for PCR2 is ~ CT value, rounding up. Minimum 7 cycles PCR2.

**PCR2**

Use dual indexed oligos from the NEBNext Multiplex Oligos for Illumina Dual Index Primers Set (E7600S). As an example:

NEBNext i501 primer (read 1) AATGATACGGCGACCACCGAGATCTACAC TATAGCCT ACACTCTTTCCCTACACGACGCTCTTCCGATCT

NEBNext i701 primer (read 2) CAAGCAGAAGACGGCATACGAGAT CGAGTAAT GTGACTGGAGTTCAGACGTGTGCTCTTCCGATCT

Or equivalent primers

**NEBNext Mix**

22.5 ul ~half of PCR1

25 ul       2x Q5 UltraII Mastermix

1.25 ul        20uM Fw primer: NEBNext i5XX primer (different for each sample)

1.25 ul        20uM Rv primer: NEBNext i7XX primer (different for each sample)

98°C |  98°C 72°C  | 72°C |    4**°** C => xx cycles (cycles from QPCR)

30sec | 10sec **1 min** | 5min |  forever

**gDNA PCR2 (final) product (406 bp)**

AATGATACGGCGACCACCGAGATCTACACNNNNNNNNACACTCTTTCCCTACACGACGCTCTTCCGATCTNNNtaccagctgccctcgTCGaCNNNNNNNNNNNNNNNNNNNNNNNNNNNNNNNNNNNAGNNNNNNNNNNNNNNNNNNNNNNNNtgattacacatatagacacgcGAGCAGCCATCTTTTATAGAATGGGtagaacccgtcctaaggactcagattgagcatcgtttgcttctcgagtactacctggtacagatgtctcttcaaacaggacggcagcgtgcagctcgccNNNNNNNNNNNNNNNgaccactaccagcagaacaccccNNNAGATCGGAAGAGCACACGTCTGAACTCCAGTCACNNNNNNNNATCTCGTATGCCGTCTTCTGCTTG

**This PCR has primer dimer and so requires gel purification. Run the 50 uL PCR2 on a 2% SYBR gel and gel purify the 406 bp band and elute in 30ul EB. Perform Tapestation and move to pooling and Nextseq sequencing.**

**Sequence a minimum of:**

**Read 1: 79 nt**

**Index 1: 8 nt**

**Read 2: 41 nt**

**Index 2: If needed, 8 nt**

**To perform cDNA prep from RNA:**

Extract total RNA from >10^7 cells using Qiagen RNeasy maxiprep kit. Elute in 150ul instead of 800ul as suggested in Maxi protocol for desired concentration.

**Reverse transcription**

First strand cDNA synthesis was performed with Invitrogen SuperscriptIII (50°C for 60 min, 70°C for 15min; cat. no. 18080085) using a reporter-RNA specific primer

070218_Citrine_RTprimer gcaactagaaggcacagtcg

with up to 15ug total RNA.

RNA – 15ug

2uM RT primer – 3ul

10mM dNTP mix – 3ul

dH2O – XX ul (up to 39 uL total volume)

incubate @ 65C for 5min and then on ice for 1min.

Add – 12ul 5x FS buffer

3ul 0.1M DTT

3ul RNaseOUT

3ul SSIII RT mix

Incubate at 50°C for 60 min, then 70°C for 10 min, then 95 deg for 5min, then 4 deg. Add 90 uL H2O, transfer to microcentrifuge tube, and nanodrop.

**To perform library prep on cDNA pre- or post-Cas9 treatment:**

**PCR1**

051018_Splice_RNA_r1seq CTTTCCCTACACGACGCTCTTCCGATCT NNN caagatccgccacaacatcg

051018_Splice_RNA_r1seq_2N CTTTCCCTACACGACGCTCTTCCGATCT NN caagatccgccacaacatcg

051018_Splice_RNA_r1seq_1N CTTTCCCTACACGACGCTCTTCCGATCT N caagatccgccacaacatcg

051018_Splice_RNA_r1seq_0N CTTTCCCTACACGACGCTCTTCCGATCT caagatccgccacaacatcg

110518_SpliceAssay_DNARNA_r2seq GGAGTTCAGACGTGTGCTCTTCCGATCT NNN ggggtgttctgctggtagtggtc

110518_SpliceAssay_DNARNA_r2seq_2N GGAGTTCAGACGTGTGCTCTTCCGATCT NN ggggtgttctgctggtagtggtc

110518_SpliceAssay_DNARNA_r2seq_1N GGAGTTCAGACGTGTGCTCTTCCGATCT N ggggtgttctgctggtagtggtc

110518_SpliceAssay_DNARNA_r2seq_0N GGAGTTCAGACGTGTGCTCTTCCGATCT ggggtgttctgctggtagtggtc

qPCR1 **TM 66**

**QPCR to determine cycle number for PCR1:**

0.2 ul cDNA

7.5 ul       2x Q5 UltraII Mastermix

0.375 ul        20uM equimolar pool of 051018_SpliceAssay_RNA_r1seq_0N-3N

0.375 ul        20uM equimolar pool of 110518_SpliceAssay_DNARNA_r2seq_ON-3N

0.75 ul 20X EvaGreen

5.8 ul    mQ (to final volume 15 ul)

98°C |  98°C   66°C  72°C  | 72°C |    4**°**C

30sec | 10sec 30sec 30sec | 5min |  forever    => 40 cycles

- Run product on gel. Expect three bands at 143, 293, and 485 bp (may all run higher than expected due to EvaGreen).
- Cycle count for PCR1 is ~ CT value, rounding up.

**Product a: cDNA PCR1 product with no splicing (485 bp)**

CTTTCCCTACACGACGCTCTTCCGATCTNNNcaagatccgccacaacatcgaggtaagttatcaccttcgtggctacagagtttccttatttgtctctgttgccggcttatatggacaagcatatcacagccatttatcggagcgcctccgtacacgctattatcggacgcctcgcgagatcaatacgattaccagctgccctcgTCGaCNNNNNNNNNNNNNNNNNNNNNNNNNNNNNNNNNNNAGNNNNNNNNNNNNNNNNNNNNNNNNtgattacacatatagacacgcGAGCAGCCATCTTTTATAGAATGGGtagaacccgtcctaaggactcagattgagcatcgtttgcttctcgagtactacctggtacagatgtctcttcaaacaggacggcagcgtgcagctcgccNNNNNNNNNNNNNNNgaccactaccagcagaacaccccNNNAGATCGGAAGAGCACACGTCTGAACTCC

**Product b: cDNA PCR1 product with unaltered splicing (293 bp)**

CTTTCCCTACACGACGCTCTTCCGATCTNNNcaagatccgccacaacatcgagAGNNNNNNNNNNNNNNNNNNNNNNNNtgattacacatatagacacgcGAGCAGCCATCTTTTATAGAATGGGtagaacccgtcctaaggactcagattgagcatcgtttgcttctcgagtactacctggtacagatgtctcttcaaacaggacggcagcgtgcagctcgccNNNNNNNNNNNNNNNgaccactaccagcagaacaccccNNNAGATCGGAAGAGCACACGTCTGAACTCC

**Product c: cDNA PCR1 product assuming exon skipping (143 bp)**

CTTTCCCTACACGACGCTCTTCCGATCTNNNcaagatccgccacaacatcgaggacggcagcgtgcagctcgccNNNNNNNNNNNNNNNgaccactaccagcagaacaccccNNNAGATCGGAAGAGCACACGTCTGAACTCC

**NEBNext Mix**

xxul **16** ug cDNA in **800 uL** PCR volume

400 ul       2x Q5 UltraII Mastermix

20 ul        20uM equimolar pool of 051018_SpliceAssay_RNA_r1seq_0N-3N

20 ul        20uM equimolar pool of 110518_SpliceAssay_DNARNA_r2seq_ON-3N

xxul    mQ dH2O (to final volume **800 ul**)

98°C   |  98°C    66°C 72°C  | 72°C |    4**°** C => xx cycles (from qPCR)

30sec  | 10sec 30sec **1 min** | 5min |  forever

**PCR purify and elute in 50ul.**

**Run 25 uL of eluate on a 2% SYBR gel (save other half as backup), isolating the region between 100-350 bp of spliced product and the region >400 bp of unspliced product. Gel purify each, eluting in 50 uL EB. The unspliced product should be treated like the gDNA after PCR1. For the spliced product:**

qPCR2 **TM 72 223-373 bp**

**QPCR to determine cycle number for PCR2:**

0.2 ul purified PCR1 product

7.5 ul       2x Q5 UltraII Mastermix

0.375 ul        20uM Fw primer: 061813_PE1

0.375 ul        20uM Rv primer: 062118_MultiplexRd2_full_noidx

0.75 ul 20X EvaGreen

5.8 ul    mQ (to final volume 20ul)

98°C |  98°C   72°C  72°C  | 72°C |    4**°**C

30sec | 10sec 30sec 30sec | 5min |  forever    => 40 cycles

These primers are unindexed versions of the primers to be used in PCR2 in order to save money and not use the valuable primers from the indexed kit:

061813_PE1 AATGATACGGCGACCACCGAGATCTACACTCTTTCCCTACACGACGCTCTTCCGATCT

062118_MultiplexRd2_full_noidx CAAGCAGAAGACGGCATACGAGAT GTGACTGGAGTTCAGACGTGTGCTCTTCCGATCT

- Run product on gel to visualize two bands at 223 and 373 bp. Not much background?
- Cycle count for PCR2 is ~ CT value, rounding up. Minimum 6 cycles PCR2.

**Product b: cDNA PCR2 product with unaltered splicing (373 bp)**

AATGATACGGCGACCACCGAGATCTACACNNNNNNNNACACTCTTTCCCTACACGACGCTCTTCCGATCT NNNcaagatccgccacaacatcgagAGNNNNNNNNNNNNNNNNNNNNNNNNtgattacacatatagacacgcGAGCAGCCATCTTTTATAGAATGGGtagaacccgtcctaaggactcagattgagcatcgtttgcttctcgagtactacctggtacagatgtctcttcaaacaggacggcagcgtgcagctcgccNNNNNNNNNNNNNNNgaccactaccagcagaacaccccNNNAGATCGGAAGAGCACACGTCTGAACTCCAGTCACNNNNNNNNATCTCGTATGCCGTCTTCTGCTTG

**Product c: cDNA PCR1 product assuming exon skipping (223 bp)**

AATGATACGGCGACCACCGAGATCTACACNNNNNNNNACACTCTTTCCCTACACGACGCTCTTCCGATCT NNNcaagatccgccacaacatcgaggacggcagcgtgcagctcgccNNNNNNNNNNNNNNNgaccactaccagcagaacaccccNNNAGATCGGAAGAGCACACGTCTGAACTCCAGTCACNNNNNNNNATCTCGTATGCCGTCTTCTGCTTG

**PCR2**

Use dual indexed oligos from the NEBNext Multiplex Oligos for Illumina Dual Index Primers Set (E7600S). As an example:

NEBNext i501 primer (read 1) AATGATACGGCGACCACCGAGATCTACAC TATAGCCT ACACTCTTTCCCTACACGACGCTCTTCCGATCT

NEBNext i701 primer (read 2) CAAGCAGAAGACGGCATACGAGAT CGAGTAAT GTGACTGGAGTTCAGACGTGTGCTCTTCCGATCT

Or equivalent primers

**NEBNext Mix**

12.5 ul PCR1

25 ul       2x Q5 UltraII Mastermix

1.25 ul        20uM Fw primer: NEBNext i5XX primer (different for each sample)

1.25 ul        20uM Rv primer: NEBNext i7XX primer (different for each sample)

10 uL dH2O

98°C |  98°C 72°C  | 72°C |    4**°** C => xx cycles (cycles from QPCR, minimum 6 cycles)

30sec | 10sec **1 min** | 5min |  forever

**Run 2 uL aliquot on gel to ensure correct size.** **PCR purify and elute in 30ul EB. Perform Tapestation and move to pooling and Nextseq sequencing.**

**Sequence a minimum of:**

**Read 1: 51 nt**

**Index 1: 8 nt**

**Read 2: 41 nt**

**Index 2: If needed, 8 nt**

1. **Building a dictionary to assign gRNAs with UMIs:**

**Use plasmid library or pre-Cas9 genomic DNA to build a dictionary tying gRNA sequences to UMIs:**

**PCR1 (Ta=63)**

Pool the following primers as a 20 uM pool (20 uL each 100 uM primer + 240 uL dH2O)

110518_SplAccUMI_r2seq_1N GGAGTTCAGACGTGTGCTCTTCCGATCT N tcaaacaggacggcagcgtg

110518_SplAccUMI_r2seq_2N GGAGTTCAGACGTGTGCTCTTCCGATCT NN tcaaacaggacggcagcgtg

110518_SplAccUMI_r2seq_3N GGAGTTCAGACGTGTGCTCTTCCGATCT NNN tcaaacaggacggcagcgtg

| 101317_U6PE1_BcX | CTTTCCCTACACGACGCTCTTCCGATCT NNNNN GGAAAGGACGAAACACCG |
| --- | --- |

**NEBNext Mix**

xxul **1ug** plasmid DNA in **100 uL** PCR volume or **8** ug gDNA in **400 uL** PCR volume

50/200 ul       2x Q5 UltraII Mastermix

2.5/10 ul        20uM equimolar pool of 110518_SplAccUMI_r2seq_1N-3N

2.5/10 ul        20uM equimolar pool of 101317_U6PE1_BcX

xxul    mQ dH2O (to final volume **100/400 ul**)

98°C   |  98°C    63°C 72°C  | 72°C |    4**°** C => 12 cycles

30sec  | 10sec 30sec **1 min** | 5min |  forever

**PCR purify and elute in 50ul.**

**gDNA PCR1 product (742 bp)**

GGAGTTCAGACGTGTGCTCTTCCGATCTNNNtcaaacaggacggcagcgtgcagctcgccNNNNNNNNNNNNNNNgaccactaccagcagaacacccccatcggcgacggccccgtgctgctgcccgacaaccactacctgagctaccagtccgccctgagcaaagaccccaacgagaagcgcgatcacatggtcctgctggagttcgtgaccgccgccgggatcactctcggcatggacgagctgtacaaggactgagactgatagtaaggcccattacctgcACCGGTNNNNNNNNNNNNNNNNNNACGCGTgcagaacacagcggttcgactgtgccttctagttgccagccatctgttgtttgcccctcccccgtgccttccttgaccctggaaggtgccactcccactgtcctttcctaataaaatgaggaaattgcatcgcattgtctgagtaggtgtcattctattctggggggtggggtggggcaggacagcaagggggaggattgggaagacaatagcaggcatgctggggatgcggtgggctctatggGTTAACTTCTAGCTCTAAAACAAAAAAGCACCGACTCGGTGCCACTTTTTcaagttgataacggacTAGCCTTATTTAAACTTGCTATGCTgtttccagcatagctcttaaacNNNNNNNNNNNNNNNNNNNNCGGTGTTTCGTCCTTTCC NNNNN AGATCGGAAGAGCGTCGTGTAGGGAAAG

qPCR **TM 72 821 bp**

**QPCR to determine cycle number for PCR2:**

0.2 ul purified PCR1

7.5 ul       2x Q5 UltraII Mastermix

0.375 ul        20uM Fw primer: 061813_PE1

0.375 ul        20uM Rv primer: 062118_MultiplexRd2_full_noidx

0.75 ul 20X EvaGreen

5.8 ul    mQ (to final volume 15 ul)

98°C |  98°C   72°C  72°C  | 72°C |    4**°**C

30sec | 10sec 30sec 30sec | 5min |  forever    => 40 cycles

These primers are unindexed versions of the primers to be used in PCR2 in order to save money and not use the valuable primers from the indexed kit:

061813_PE1 AATGATACGGCGACCACCGAGATCTACACTCTTTCCCTACACGACGCTCTTCCGATCT

062118_MultiplexRd2_full_noidx CAAGCAGAAGACGGCATACGAGAT GTGACTGGAGTTCAGACGTGTGCTCTTCCGATCT

- Run product on gel to visualize 821 bp band. Not much background?
- Cycle count for PCR2 is ~ CT value, rounding up. Minimum 6 cycles PCR2.

**PCR2**

Use dual indexed oligos from the NEBNext Multiplex Oligos for Illumina Dual Index Primers Set (E7600S). As an example:

NEBNext i501 primer (read 1) AATGATACGGCGACCACCGAGATCTACAC TATAGCCT ACACTCTTTCCCTACACGACGCTCTTCCGATCT

NEBNext i701 primer (read 2) CAAGCAGAAGACGGCATACGAGAT CGAGTAAT GTGACTGGAGTTCAGACGTGTGCTCTTCCGATCT

Or equivalent primers

**NEBNext Mix**

22.5 ul ~half of PCR1

25 ul       2x Q5 UltraII Mastermix

1.25 ul        20uM Fw primer: NEBNext i5XX primer (different for each sample)

1.25 ul        20uM Rv primer: NEBNext i7XX primer (different for each sample)

98°C |  98°C 72°C  | 72°C |    4**°** C => xx cycles (cycles from QPCR)

30sec | 10sec **1 min** | 5min |  forever

**gDNA PCR2 (final) product (821 bp)**

AAGCAGAAGACGGCATACGAGATNNNNNNNNGTGACTGGAGTTCAGACGTGTGCTCTTCCGATCT NNNtcaaacaggacggcagcgtgcagctcgccNNNNNNNNNNNNNNNgaccactaccagcagaacacccccatcggcgacggccccgtgctgctgcccgacaaccactacctgagctaccagtccgccctgagcaaagaccccaacgagaagcgcgatcacatggtcctgctggagttcgtgaccgccgccgggatcactctcggcatggacgagctgtacaaggactgagactgatagtaaggcccattacctgcACCGGTNNNNNNNNNNNNNNNNNNACGCGTgcagaacacagcggttcgactgtgccttctagttgccagccatctgttgtttgcccctcccccgtgccttccttgaccctggaaggtgccactcccactgtcctttcctaataaaatgaggaaattgcatcgcattgtctgagtaggtgtcattctattctggggggtggggtggggcaggacagcaagggggaggattgggaagacaatagcaggcatgctggggatgcggtgggctctatggGTTAACTTCTAGCTCTAAAACAAAAAAGCACCGACTCGGTGCCACTTTTTcaagttgataacggacTAGCCTTATTTAAACTTGCTATGCTgtttccagcatagctcttaaacNNNNNNNNNNNNNNNNNNNNCGGTGTTTCGTCCTTTCC NNNNN AGATCGGAAGAGCGTCGTGTAGGGAAAGAGTGTNNNNNNNNGTGTAGATCTCGGTGGTCGCCGTATCATT

**Run 2 uL aliquot on gel to ensure 581 bp band.** **PCR purify and elute in 30ul EB. Perform Tapestation and move to pooling and Nextseq sequencing.**

**Sequence a minimum of:**

**Read 1: 47 nt**

**Index 1: 8 nt**

**Read 2: 40 nt**

**Index 2: If needed, 8 nt**

**Sequencing analysis instructions**

**For genomic DNA samples and unspliced RNA samples**

Read 1 begins with 0-3 randomized bases to mitigate monotemplate issues, then proceeds to a constant primer, followed by the variable splice-acceptor insert sequence. It is probably easiest to align to and then remove the constant primer sequence prior to analysis.

Read 1:

NNNtaccagctgccctcgTCGaCNNNNNNNNNNNNNNNNNNNNNNNNNNNNNNNNNNNAGNNNNNNNNNNNNNNNNNNNNNNNNtgattacacatatagacacgcGAGCAGCCATCTTTTATAGAATGGGtagaacccgtcctaaggactcagattgagcatcgtttgcttctcgagtactacctgg

Read 2 begins with 0-3 randomized bases to mitigate monotemplate issues, then proceeds to a constant primer, followed by the UMI associated with each splice-acceptor. There should be a large but discrete number of UMIs associated with each splice-acceptor, but importantly each UMI should only be associated with a single splice-acceptor sequence because of the vast excess of possible 15-nt UMIs. It is probably easiest to align to and then remove the constant primer sequence prior to analysis.

NNNggggtgttctgctggtagtggtcNNNNNNNNNNNNNNNggcgagctgcacgctgccgtcctgtttgaagagacatctgtaccaggtagtactcgagaagcaaacgatgctcaatctgagtccttaggacgggttctaCCCATTCTATAAAAGATGGCTGCTCgcgtgtctatatgtgtaatca

a

**For spliced RNA samples:**

There are two likely predominant product types from these samples, and there are likely also additional product types that don’t neatly fit into either of these categories.

**Read 1 for these two predominant products will look like:**

**Product b: product with unaltered splicing**

NNNcaagatccgccacaacatcgagAGNNNNNNNNNNNNNNNNNNNNNNNNtgattacacatatagacacgcGAGCAGCCATCTTTTATAGAATGGGtagaacccgtcctaaggactcagattgagcatcgtttgcttctcgagtactacctggt

**Product c: product with perfect exon skipping**

NNNcaagatccgccacaacatcgaggacggcagcgtgcagctcgccgaccactaccagcagaacacccccatcggcgacggccccgtgctgctgcccgacaaccactacctgagctaccagtccgccctgagcaaagaccccaacgagaa

**Note that there may be other products where cryptic splice-acceptors have appeared.**

Read 2 begins with 0-3 randomized bases to mitigate monotemplate issues, then proceeds to a constant primer, followed by the UMI associated with each splice-acceptor. It is probably best to use UMI information from the “dictionary” to assign reads to their presumed splice-acceptor and then use read 1 to determine their splicing outcome.

NNNggggtgttctgctggtagtggtcNNNNNNNNNNNNNNNggcgagctgcacgctgccgtcctgtttgaagagacatctgtaccaggtagtactcgagaagcaaacgatgctcaatctgagtccttaggacgggttctaCCCATTCTATAAAAGATGGCTGCTCgcgtgtctatatgtgtaatca

**For gRNA/UMI dictionary samples:**

The goal of this sequencing type is to pair gRNAs with UMIs. In the plasmid/gDNA sequencing previously, target sequences were paired with UMIs; however, multiple gRNAs may be paired with the same target, so it is helpful to disambiguate them using the UMI as the common link. Note that it is not possible to sequence the gRNA, target, and UMI in the same sequencing reaction due to the distance between them.

Read 1 for these samples contains information on the gRNA. It begins with a staggered barcode (to avoid monotemplate) to allow multiplexing followed by a common primer that all reads should match to:

Example beginning of read 1 with BcA: ACTAG GGAAAGGACGAAACACCG

After this sequence comes the variable 19-20 nt gRNA. Note that in this format, the beginning G from all gRNAs is in the above sequence, so this sequence should not be included in the variable gRNA to be mapped.

Read 2 begins with 1-3 randomized bases to mitigate monotemplate issues, then proceeds to a constant primer, followed by the UMI associated with each splice-acceptor/gRNA pair. This UMI will help to build a dictionary with which to map splicing outcomes.

Read 2: NNNtcaaacaggacggcagcgtgcagctcgccNNNNNNNNNNNNNNNgaccactaccagcagaacacccc
