# Supplementary material for: Machine learning based CRISPR gRNA design for therapeutic exon skipping
Source: PLoS Comput Biol. 2021 Jan 8;17(1):e1008605. doi: 10.1371/journal.pcbi.1008605 (PMC7819613; doi:10.1371/journal.pcbi.1008605)
Supplement: Attachment — Submitted filename: Reproducible_report_PCOMPBIOL_D_20_01290R1 (1).pdf [file pcbi.1008605.s021.pdf]

Reproducibility report for: Machine learning based CRISPR gRNA design for therapeutic exon skipping.
Submitted to: PLoS Computational Biology
Manuscript number/identifier: PCOMPBIOL-D-20-01290R1

Curation outcome summary: Successfully reproduced the results as described in the manuscript.

Box 1: Criteria for repeatability and reproducibility

- [x] **Model source code provided:**
  - [x] Source code: a standard procedural language is used (e.g. MATLAB, Python, C)
    - [x] There are details/documentation on how the source code was compiled
    - [x] There are details on how to run the code in the provided documentation
    - [x] The initial conditions are provided for each of the simulations
    - [x] Details for creating reported graphical results from the simulation results
  - [ ] Source code: a declarative language is used (e.g. SBML, CellML, NeuroML)
    - [ ] The algorithms used are defined or cited in previous articles
    - [ ] The algorithm parameters are defined
    - [ ] Post-processing of the results are described in sufficient detail
- [ ] **Executable model provided:**
  - [ ] The model is executable without source (e.g. desktop application, compiled code, online service)
    - [ ] There are sufficient details to repeat the required simulation experiments
- [x] **The model is described mathematically in the article(s):**
  - [x] Equations representing the biological system
  - [ ] There are tables or lists of parameter values
  - [ ] There are tables or lists of initial conditions
  - [ ] Machine-readable tables of parameter values
  - [ ] Machine-readable tables of initial conditions
- [ ] **The simulation experiments using the model are described mathematically in the article:**
  - [ ] Integration algorithms used are defined
  - [ ] Stochastic algorithms used are defined
  - [ ] Random number generator algorithms used are defined
  - [ ] Parameter fitting algorithms are defined
  - [ ] The paper indicates how the algorithms yield the desired output

Box 2: Criteria for accessibility

- ■ Model/source code is available at a public repository or researcher's web site
  - ☐ Prohibitive license provided
  - ☐ Permissive license provided
  - ■ Open-source license provided
- ☐ All initial conditions and parameters are provided
- ☐ All simulation experiments are fully defined (events listed, collection times and measurements specified, algorithms provided, simulator specified, etc.)

Box 3: Rules for Credible practice of Modeling and Simulation[a]

[a]Model credibility is assessed using the Interagency Modeling and Ananlysis Group conformance rubric: https://www.imagwiki.nibib.nih.gov/content/10-simple-rules-conformance-rubric

- ■ Define context clearly: Extensive
- ■ Use appropriate data: Extensive
- ■ Evaluate within context: Extensive
- ☐ List limitations explicitly: Insufficient
- ■ Use version control: Extensive
- ■ Document adequately: Extensive
- ☐ Conform to standards: Insufficient

Box 4: Evaluation

- ■ Model and its simulations could be repeated using provided declarative or procedural code
- ■ Model and its simulations could be reproduced

Summary comments: We appreciate the provision by the authors of downloadable model source codes via the Github links (https://github.com/gifford-lab/skipguide, https://github.com/gifford-lab/skipguide-analysis). These were used in our assessment of the reproducibility of the results presented in the manuscript. We successfully ran the Jupyter notebooks using the pre-computed files as described in the Github repository to reproduce the results as described in the manuscript.

Anand K. Rampadarath[1], PhD
Curator
Center for Reproducible Biomedical Modeling

David P. Nickerson, PhD
Curation Service Director
Center for Reproducible Biomedical Modeling

Auckland Bioengineering Institute,
University of Auckland

[1] Email: a.rampadarath@auckland.ac.nz
