# Supplementary material for: Machine learning based CRISPR gRNA design for therapeutic exon skipping
Source: PLoS Comput Biol. 2021 Jan 8;17(1):e1008605. doi: 10.1371/journal.pcbi.1008605 (PMC7819613; doi:10.1371/journal.pcbi.1008605)
Supplement: Attachment — Submitted filename: Response 111120.docx [file pcbi.1008605.s020.docx]

-------- Forwarded Message --------

Subject:	Decision from PLOS Computational Biology (PCOMPBIOL-D-20-01290) - [EMID:884be3d63de51a2a]

Date:	7 Sep 2020 11:07:50 -0400

From:	PLOS Computational Biology <em@editorialmanager.com>

Reply-To:	PLOS Computational Biology <ploscompbiol@plos.org>

To:	David Gifford <gifford@csail.mit.edu>

Dear Dr. Gifford,

Thank you very much for submitting your manuscript "Machine learning based CRISPR gRNA design for therapeutic exon skipping" for consideration at PLOS Computational Biology.

As with all papers reviewed by the journal, your manuscript was reviewed by members of the editorial board and by several independent reviewers. In light of the reviews (below this email), we would like to invite the resubmission of a significantly-revised version that takes into account the reviewers' comments. In particular, more than one reviewer raised the issue of accessibility of the source code, as well as the validation of the mESC prediction results. Please ensure that these concerns are thoroughly addressed in the revised manuscript and response letter.

We cannot make any decision about publication until we have seen the revised manuscript and your response to the reviewers' comments. Your revised manuscript is also likely to be sent to reviewers for further evaluation.

When you are ready to resubmit, please upload the following:

[1] A letter containing a detailed list of your responses to the review comments and a description of the changes you have made in the manuscript. Please note while forming your response, if your article is accepted, you may have the opportunity to make the peer review history publicly available. The record will include editor decision letters (with reviews) and your responses to reviewer comments. If eligible, we will contact you to opt in or out.

[2] Two versions of the revised manuscript: one with either highlights or tracked changes denoting where the text has been changed; the other a clean version (uploaded as the manuscript file).

Important additional instructions are given below your reviewer comments.

Please prepare and submit your revised manuscript within 60 days. If you anticipate any delay, please let us know the expected resubmission date by replying to this email. Please note that revised manuscripts received after the 60-day due date may require evaluation and peer review similar to newly submitted manuscripts.

Thank you again for your submission. We hope that our editorial process has been constructive so far, and we welcome your feedback at any time. Please don't hesitate to contact us if you have any questions or comments.

Sincerely,

Wei Li, Ph.D.

Guest Editor

PLOS Computational Biology

Jian Ma

Deputy Editor

PLOS Computational Biology

***********************

Reviewer's Responses to Questions

Comments to the Authors:

Please note here if the review is uploaded as an attachment.

Reviewer #1: Reproducibility report has been uploaded as an attachment.

Need to provide source code to permit reproducibility. Note their analysis -

We apologize for the oversight, but we forgot to make the GitHub repositories public. The links should be publicly accessible now:

<https://github.com/gifford-lab/skipguide-analysis> (contains the code and instruction for producing the results of this submission)

<https://github.com/gifford-lab/skipguide> (contains a readily usable and open source implementation of SkipGuide)

The sequencing reads have been deposited to NCBI Sequence Read Archive (SRA) under BioProject accession PRJNA647416, and will be made public upon publication. The review link is available at <https://dataview.ncbi.nlm.nih.gov/object/PRJNA647416?reviewer=7desib600hdd588j8s5kias2b1&archive=sra>

Together with the provided supplementary tables and data with this submission, we believe everything necessary for reproducibility is now available.

Reviewer #2: Deleterious Exon skipping strategy is effective to restore gene function and potentially cure some kinds of genetic disorders. Previous antisense oligo-based treatment for exon skipping requires lifespan delivery of drugs which are troublesome and expensive to the affected family. CRIPSR-Cas9 or other related Genome editing technique provide a potentially permanent solution to alleviate the symptom of a patient. However, there is no specific tool available to screen the efficient guide RNA that leads to frequent skipping of target exons holding pathogenic variants. The authors present two novel contributions to the gene therapy field with genome editing. They first develop a cell type specific genome integrated library called lib-SA as an extension of lib-A in inDelphi and preprocessing pipeline of the sequence data to quantify the Cas9 template-free repairing genotype outcome of the deleterious splicing junctions and associated exon-skipping frequency. They also provide a modular framework SkipGuide that combines the state-of-art inDelphi and MMSplice to predict the splice-in percent with a high precise performance. The library and the computational framework jointly extend the genome editing to provide an generalized therapeutic strategy to treat multiple genetic disorders.

Here are my comments for the manuscript to address the points that might not be sufficiently clear or need to be improved.

Major comments:

1. The introduction section mentioned five good examples of genetic disorder that would be hopefully cured by exon skipping. In general, how many genetic disorders so far could be potentially cured by exon skipping strategy? This information might be obtained by adding disease annotation in S1 Table, what are the diseases for the corresponding candidate pathogenic genes and sgRNA?

In general, the number of diseases potentially treatable by exon skipping therapy is unknown. As described in the Methods section of our manuscript (lines 302 to 312 of the revised text), we applied a narrow set of constraints on the HGMD, Ensembl, and Pfam databases to identify 1,927 exons in 923 unique genes with known pathogenic indels potentially amenable to CRISPR-Cas9 (acceptor site ablation) based exon skipping treatment. These exons are described in the S1 Table under the “gene_symbol”, “chromosome”, and “exon_start” columns.

We are unable to provide further HGMD annotations such as disease names or phenotype due to HGMD conditions of use (http://www.hgmd.cf.ac.uk/docs/copyright.html). However, those with access to HGMD can obtain these annotations themselves by searching the gene symbols and corroborating the “exon_start” positions provided in S1 Table. We also updated the description of S1 Table with more useful information on specific columns (lines 692 to 697 of the revised text): ‘The “Designed 61-bp target site (37i-24e, AG)” column contains the designed target sequences. The “Designed gRNA (NGG orientation, 19 and 20)” column contains the gRNA sequences. The “exon_start” column contains positions relative to the Genome Reference Consortium Human Build 37 (GRCh37).

2. In line 246, exon A and donor site are fixed, and SkipGuide ignore the donor site contribution in the predictive pipeline, would the acceptor site effect more important than donor site in the library design principal? Or is it a simplified library which could be further extended to study the donor site sequence effect?

We focused on the acceptor site effects because acceptor site alteration allows reliable control over which exon is skipped. To illustrate, consider the following primary RNA transcript (where D and A denote donor and acceptor sites respectively):

[Exon1]D1---A2[Exon2]D2---A3[Exon3]D3---A4[Exon4]

The introns recognized by the splicing machinery would primarily be {D1---A2, D2---A3, D3---A4}, which results in the spliced RNA transcript [Exon1][Exon2][Exon3][Exon4]. Let’s consider the goal of skipping Exon3.

If just A3 is completely ablated, then we primarily expect the introns recognized by the splicing machinery to be {D1---A2, D2---A4}, which results in the desired spliced transcript [Exon1][Exon2][Exon4]. The minor product [Exon1][Exon2D2---Exon3][Exon4] may be possible if introns {D1---A2, D3---A4} are recognized instead due to alternative splicing.

If just D3 is completely ablated, however, we primarily expect the introns to be {D1---A2, D2---A3}, which results in the undesired spliced transcript [Exon1][Exon2][Exon3---A4Exon4] with Exon3 retained. The minor product [Exon1][Exon2][Exon4] may be possible if introns {D1---A2, D2---A4} are recognized instead due to alternative splicing.

Some previous studies have employed donor site ablation (Min et al., 2019), and thus donor site ablation is a potential future extension to our work. However, most previous studies on CRISPR induced exon skipping only employ acceptor site ablation (Li et al., 2015, Amoasii et al., 2018, Chen et al., 2018, Min et al., 2019). Our present work is focused on developing a predictive tool for guiding such studies.

We’ve updated our discussion section with the above point; please refer to lines 263 to 269 of the revised manuscript.

Response references:

- Li, Hongmei Lisa, et al. "Precise correction of the dystrophin gene in duchenne muscular dystrophy patient induced pluripotent stem cells by TALEN and CRISPR-Cas9." Stem cell reports 4.1 (2015): 143-154.
- Amoasii, Leonela, et al. "Gene editing restores dystrophin expression in a canine model of Duchenne muscular dystrophy." Science 362.6410 (2018): 86-91.
- Chen, Dafeng, et al. "CRISPR/Cas9-mediated genome editing induces exon skipping by complete or stochastic altering splicing in the migratory locust." BMC biotechnology 18.1 (2018): 1-9.
- Min, Yi-Li, et al. "CRISPR-Cas9 corrects Duchenne muscular dystrophy exon 44 deletion mutations in mice and human cells." Science advances 5.3 (2019): eaav4324.

3. In Figure 2C, is the occurrence of AG motif in the acceptor site have any effect on the prediction? Which of the sgRNA-target pairs and associated diseases could have the best correlation between observed and prediction by SkipGuide? What the prediction correlation histogram separately for deletion and 1-bp insertion genotype since the two genotypes uses different models? In Fig. 2D, an associated scatterplot may be better to show the prediction consistency with observed frequency.

We don’t believe the occurrence of the AG motif in the acceptor site has any significant effects on inDelphi’s prediction. In our revised manuscript (lines 111 to 120, and S2 FigA), we showed that the set of sequences used to train inDelphi (Shen et al., 2018) do not overlap, and are not homologous to our lib-SA sequences that make up dat-A. Thus, our sequences (with the AG motifs) are “unseen” by inDelphi training, and the high correlation between observed and predicted repairs in Fig 2C shows that inDelphi is generalizing well to our sequences. Furthermore, inDelphi is originally trained and validated on both artificial genome-integrated and natively endogenous sequences ranging in sequence composition, microhomology strength, and heterogeneity of indel outcomes, and is thus meant to generalize well on any sequence.

We induce a scatter plot as suggested to characterize prediction consistency with actual performance as Fig 4. Fig 4 shows the actual vs SkipGuide predicted percent spliced-in values of our set of sgRNA-target pairs, and Fig 4 includes evaluation metrics such as correlation and mean square error. The performance of SkipGuide on a particular disease of interest will depend upon many factors, and thus it may not generalize well beyond our test set where we may have few examples for a given disease. We instead report aggregate performance across all diseases as we have sufficient support for these conclusions.

We agree that showing the prediction correlation histogram separately for deletion and 1-bp insertion is a good idea. Please refer to S2 Fig and lines 128 to 138 in the revised text for this revision. We found that when evaluated in isolation, the median correlation between actual and predicted 1-bp insertion is 0.95, and the median correlation between actual and predicted deletions is 0.75.

We imagine a scatterplot version of Fig 2D would be an actual vs predicted frequencies plot, where the two axes range between 0 and 1, and many points showing up on the diagonal would demonstrate consistency between actual and predicted frequencies. However, it’s unclear to us how the genotype information would be displayed on such a scatterplot. Our intention for showing Fig 2D is to show the reader visually an example of what inDelphi is predicting (genotypes and frequencies), so we feel keeping Fig 2D as is would be most beneficial to the reader’s understanding.

Response references:

- Shen, Max W., et al. "Predictable and precise template-free CRISPR editing of pathogenic variants." Nature 563.7733 (2018): 646-651.

4. In Fig.3.group 10-fold cross validation may have variable performance for different sampling, multiple repeats of group 10-fold cross validation might be considered to evaluate the model robustness.

Thanks for this suggestion. We’ve included extra analyses that repeat 10-fold cross validation 1,000 times (each with a randomly sampled folds split), shown as S10 Fig. We found that all three models (wMMSplice, MetaSplice, and SkipGuide) are quite robust to varying fold splits--the results of performing 10-fold cross validation once (Table 1 and Fig 4A) are very similar to those of repeating 10-fold cross validation 1,000 times (S10 Fig).

5. A clinical sgRNA would own high efficiency of exon skipping without off-target effect, what about the off-target activity from the top ranked sgRNA by SkipGuide? Is off-target activity correlated with exon-skipping frequency?

We believe off-target activities are unrelated to exon-skipping frequency, but it’s not possible for us to give a general or principled statement about the properties of these gRNAs; the experimental setup presented in our study does not measure these effects. Downstream analyses of true off/on target effects are necessary. In practice, it’s also possible to use predictive tools such as Azimuth (Doench et al., 2016) and Elevation (Listgarten et al., 2018) to produce a set of gRNAs with predicted minimal off-target and maximal on-target activities respectively before using SkipGuide and performing downstream experimental screening.

We’ve enhanced our discussion with some of these points. Please refer to lines 286 to 292 of our revised manuscript.

Response references:

- Doench, John G., et al. "Optimized sgRNA design to maximize activity and minimize off-target effects of CRISPR-Cas9." Nature biotechnology 34.2 (2016): 184-191.
- Listgarten, Jennifer, et al. "Prediction of off-target activities for the end-to-end design of CRISPR guide RNAs." Nature biomedical engineering 2.1 (2018): 38-47.

6. For the top predicted sgRNA with highest exon-skipping frequency (lowest skip-in percent) in lib-SA, what's the efficiency of exon skipping in the real transcriptomics of mESC cell line? It would be valuable to validate a few top predicted sgRNA in real exon skipping of the transcriptomics since the exon skipping would be also affected by chromatin state (H3K36me3), donor site, or other sequence context which are not considered in lib-SA.

We have independently demonstrated in a previous work that Cas9 is a pioneer factor, i.e. its ability to modify DNA is unaffected by chromatin state (Barkal et al., 2016). Our high throughput experiments in this paper validates the use of gRNAs with Cas9 to ablate genome-integrated sequences (dat-A) that leads to changes in exon skipping levels (dat-B) in their native cellular context. We have previously validated that such high-throughput assay results match edits at endogenous loci in our work on inDelphi (Shen et al., 2018). The inDelphi paper also details experiments that show Cas9 coupled with gRNAs selected using inDelphi’s predictions led to efficient repair of microduplication alleles endogenously, including correction in primary patient-derived fibroblasts of pathogenic alleles to wild-type genotype for Hermansky–Pudlak syndrome and Menkes disease (Shen et al., 2018).

Therefore, we expect that CRISPR-Cas9 mediated acceptor sequence disruption to cause changes in exon skipping levels will work endogenously, but there is always a need to characterize how well it works in specific cases. In practice SkipGuide would be useful for identifying new places that can work well, and downstream high throughput experiments are necessary to characterize how well they work specifically.

We have incorporated some of these points in our discussion section of the revised manuscript, lines 278 to 285.

Response references:

- Barkal, Amira A., et al. "Cas9 functionally opens chromatin." PLoS One 11.3 (2016): e0152683.
- Shen, Max W., et al. "Predictable and precise template-free CRISPR editing of pathogenic variants." Nature 563.7733 (2018): 646-651.

7. In the SkipGuide pipeline, are the weights fixed for inDelphi and fine-tuned for wMMSplice?

Yes, inDelphi is fixed and used as is, and wMMSplice is tuned with our experimental data. To be clear, the underlying MMSplice models are also fixed. wMMSplice is a ridge regression model (followed by a sigmoid transformation) that combines the predictions from the intronic, acceptor site, and exonic prediction modules of the MMSplice framework. Only the regression model is fitted with our experimental data.

We have significantly updated our Methods section of the revised manuscript to be clearer on this point (see lines 515 to 538). Note that due to a suggestion by another reviewer to try out SpliceAI (Jaganathan et al., 2019), we’ve revised the paper to include analyses on another method we call MetaSplice. MetaSplice is just like wMMSplice, except SpliceAI predictions are additionally incorporated into the linear model (see lines 540 to 554). Thus, the above answer to your question also applies to MetaSplice.

Response references:

- Jaganathan, Kishore, et al. "Predicting splicing from primary sequence with deep learning." Cell 176.3 (2019): 535-548.

8. For the perturbation study, what would be the precision and recall after ablating inDelphi using a similar threshold as before ablating inDelphi? What’s the performance of inDelphi model trained on another cell line other than mESC? inDelphi model with specific cell line could increase 0.07 prediction correlation, what would the performance be if the inDelphi fine-tuned on dat-A? What about the prediction correlation if the wMMSplice output is replaced with a constant 1 while only use the inDelphi model on mESC?

As per your suggestions, we’ve included precision and recall analyses (and ROC AUC as suggested by another reviewer) for the perturbation study (see S8 Fig of the revised manuscript). As shown in the figure, when inDelphi is ablated, SkipGuide’s precision is 0.7 and recall is 0.37 at the 0.3 threshold, and precision is 0.43 and recall 0.79 at the 0.5 threshold.

The original inDelphi paper (Shen et al., 2018) reported their performance of the inDelphi model trained on data from various cell types other than mESC (U2OS, HEK293, HCT116, K562), and showed that cell type specific models are necessary for good accuracy. Our experimental data is from mESC, so we used the inDelphi model trained on mESC for evaluation. While fine tuning on dat-A would allow inDelphi to be more accurate on dat-A, inDelphi is originally trained and validated on both artificial genome-integrated and natively endogenous sequences ranging in sequence composition, microhomology strength, and heterogeneity of indel outcomes, and is thus more generalized (Shen et al., 2018). In our revised manuscript, we also showed that inDelphi, despite not fine-tuned on dat-A and is trained on sequences very different from (and more diverse than) that of dat-A (see S2 FigA of the revised manuscript), generalizes well in predicting our observed repair genotypic frequency distribution in dat-A. This also ensures a very effective train/test split for inDelphi.

We’ve added the requested perturbation analysis where vMMSplice output is replaced by 1 in S8 FigG of the revised manuscript. The prediction correlation is undefined if MetaSplice (instead of wMMSplice, though the result would still be the same) output is replaced with a constant 1, because then SkipGuide predictions will always be 1, and we get a vertical line in the actual vs predicted relationship.

Response references:

- Shen, Max W., et al. "Predictable and precise template-free CRISPR editing of pathogenic variants." Nature 563.7733 (2018): 646-651.

9. In line 382, what kind of linear model for wMMSplice, is it logistic regression model for classification? Does the model use l1 or l2 penalty for avoiding possibly colinear genotype features? How to interpret the model based on rule of the splice acceptor target sequence or sgRNA sequence composition?

As stated in line 382 of the original text, wMMSplice is a linear regression model (followed by a sigmoid transformation) that combines the predictions from the intronic, acceptor site, and exonic prediction modules of the MMSplice framework (see also Fig 1B). Only the linear regression model is fitted with our experimental data; the underlying MMSplice models are fixed. Each of the MMSplice modules outputs a predicted logit(PSI) value given a repair genotype, and wMMSplice combines them into a single logit(PSI) prediction, followed by a sigmoid transform to yield a final PSI prediction ranging between 0 and 1.

We agree these points should be made much more explicit, so we’ve added more details on our models in our Methods section (lines 515 to 554 in the revised manuscript), as well as in the main text (lines 160 to 189 in the revised manuscript). In the original manuscript, wMMSplice uses the classic linear least square regression without regularization. We’ve updated our analysis to instead use ridge regression (linear least squares with L2 regularization) for wMMSplice as well as for MetaSplice.

To reiterate, wMMSplice and MetaSplice are meta models that combine predictions from the three MMSplice prediction modules (plus SpliceAI predictions in the case of MetaSplice). Thus, for interpretability of genotype features, one would need to analyze the underlying MMSplice and SpliceAI (deep learning) models; we feel this type of analysis falls outside the scope of this study.

10. The most advantage of CRIPSR-Cas9 over antisense oligo treatment is the permanent treatment effect. In lib-SA, Cas9 transfects the mESC with Blasticidin selection to maximize Cas9 activity. How would the Cas9 be delivered to maximize its activity in the long term for a clinical setting? This might be discussed in the discussion section to provide a practical insight of using lib-SA and SkipGuide.

Delivery approaches for CRISPR-Cas9 therapeutics is an area of active research today, and significant advances have been made in viral (using lentiviral, adeno-associated viral, or adenoviral vectors (Wilbie et al., 2019)), mRNA-based, plasmid-based, and protein based delivery approaches (Luther et al., 2018). However, SkipGuide is meant to be a tool for early exploration, and we feel discussing clinical delivery of CRISPR-Cas9 therapeutics may be out of the scope of this manuscript. We have instead updated our discussion section to include more points on SkipGuide limitations in practice; please refer to lines 240 to 297 in our revised manuscript.

Response references:

- Wilbie, Danny, Johanna Walther, and Enrico Mastrobattista. "Delivery aspects of CRISPR/Cas for in vivo genome editing." Accounts of chemical research 52.6 (2019): 1555-1564.
- Luther, D. C., et al. "Delivery approaches for CRISPR/Cas9 therapeutics in vivo: advances and challenges." Expert opinion on drug delivery 15.9 (2018): 905-913.

Minor comments:

• Full name of MMSplice should be written as modular modeling of splicing, and the full name for wMMSplice should be also listed in the main text.

Thank you for catching this. Please refer to lines 36 to 41 in the revised manuscript for this correction.

• In Fig 1A and S1 Fig, it would be better to annotate the dat-A and dat-B sequences on the workflow schematics.

Thank you for this suggestion. We have updated Fig 1A and S1 Fig with dat-A and dat-B annotations.

• In Fig.1 and S1 Fig, there is no intron between exon B and C in lib-SA plasmid, would exon C be skipped as well in RNA splice sequence?

Thank you for pointing out that this can be made clearer. No, only Exon B would be skipped. There’s an alternative splice acceptor between Exon B and Exon C, so if the splice acceptor between Exon A and Exon B is ablated, then the alternative splice acceptor is used instead such that Exon A and Exon C are spliced together, leading to Exon B skipping. This alternative splicing is shown in the “RNA Primary” block of Fig 1A and S1A Fig. S1 Text (referenced at the beginning of the Results section) shows the alternate acceptor AG at the end of the Exon B sequence, and a detailed annotation of this sequence construct is available in the first page of S3 Text (referenced in the Library cloning section of Methods).

We have updated the text to clarify this point. Please refer to lines 67 to 72 in the revised manuscript.

• In benchmark dat-B, only MMSplice and MaxEntScan are evaluated, how about other method mentioned in the introduction, e.g. SPANR?

In the original manuscript, we evaluated MaxEntScan as a baseline comparison with MMSplice, but we did not evaluate SPANR, COSSMO, or HAL because as mentioned in the introduction and results (lines 44 to 49 and lines 141 to 146 in the revised manuscript), the more recently published MMSplice (Cheng et al., 2019) demonstrated superior performance over these models. However, as suggested by another reviewer, we’ve evaluated another recent method called SpliceAI (Jaganathan et al., 2019), which has not been directly compared with MMSplice before.

In the revised manuscript, we present comparisons between MaxEntScan, SpliceAI, MMSplice, and the two metamodels we’ve built, wMMSplice and MetaSplice (refer to Table 1 and Fig 3, and the results text in lines 141 to 189). As in the original manuscript, wMMSplice is a linear model that combines the predictions from the three MMSplice prediction modules. MetaSplice in the revised manuscript is just like wMMSplice, except SpliceAI predictions are also included in the linear model (refer to lines 515 to 554 in Methods).

Response references:

- Cheng, Jun, et al. "MMSplice: modular modeling improves the predictions of genetic variant effects on splicing." Genome biology 20.1 (2019): 1-15.
- Jaganathan, Kishore, et al. "Predicting splicing from primary sequence with deep learning." Cell 176.3 (2019): 535-548.

• In line 110, the main text description is not consistent with the Fig. 2B, Fig. 2B show the observed frequency of inserted based at -4 bp, however, the text describes the consistency of the predicted frequencies and observed frequency.

Thank you for catching this. For predicting 1-bp insertions, inDelphi employs a nearest neighbors model that assumes genotypes of 1-bp insertions are local sequence context dependent, and are predominantly duplications of the -4 nucleotide from the NGG SpCas9 PAM. Our intention was to show in Fig 2B that this inductive bias generalizes to the 1-bp insertions observed in dat-A (which indirectly suggests that the nearest neighbor model should work quite well). However, we agree that we should also directly evaluate 1-bp insertion predictions. In the revised manuscript, we demonstrated that inDelphi’s 1-bp insertion predictions highly correlate with our observed 1-bp insertions in dat-A (r = 0.95). Please refer to lines 128 to 138, and S2 Fig of the revised manuscript.

• In line 162, after binarizing the observed skip-in values, what would be ROC curve and the Area Under the Curve (AUC) look like?

Thank you for this suggestion, we’ve augmented Fig 4 to show the ROC curve and AUC values for each fold of cross validation. By binarizing the empirically observed percent spliced-in values using a threshold of 0.5, we found that SkipGuide (using MetaSplice internally) predictions yield a mean AUC of 0.84 (0.03 standard deviation) using 10 fold cross validation. Repeating the 10-fold cross validation 1,000 times also yields a mean AUC of 0.84 (0.04 standard deviation), and we’ve included this information in S10 Fig of the revised manuscript.

• In later library design section from Methods, which version of HGMD, Ensembl and Pfam were used?

Thank you for catching this. We have updated the text with the versions (HGMD Professional release 2018.1, Emsembl release 92, Pfam release 31). Please refer to lines 302 to 309 of the revised manuscript.

• How are the sgRNA on-target threshold of BOTM and Azimuth decided for designing lib-SA?

Thank you for mentioning this, because we realized a mistake in our wording of line 231 in the original submission. It originally reads “(Azimuth score above 0.2 or BOTM score below 0.65)”, but it should actually read “(Azimuth score above 0.2 and BOTM score above 0.65)”. This correction has been made in the revised submission as line 319.

These thresholds were chosen manually. We ultimately want to retain gRNAs that will efficiently cut and induce non-wild-type repair outcomes (high on-target activity). Azimuth predicts a given gRNA’s ability to knock out a target gene, but it does not directly predict the frequency of non-wild type repair frequency. We reason that the frequency of protein knockdown as predicted by Azimuth depends on the rate of non-wild-type repair, and the rate of frameshift repair out of all non-wild-type repair outcomes. That is, even if Azimuth predicts a given gRNA has low efficiency in knocking out a protein, it may be because the gRNA would induce high rates of non-wild-type repair outcomes that do not impair the reading frame. Due to this concern, we used a minimum threshold Azimuth score (we picked 0.2) to first filter out gRNAs predicted to very likely have low on-target activity. We then developed and used BOTM for the primary prediction of high on-target gRNAs, as described in S2 Text. The 0.65 threshold for BOTM is manually determined on held-out data.

To make these points clearer, we’ve augmented S2 Text with the above information.

• Methods on preprocessing lib-SA to get repaired genotype outcome and splice-in percent are the key part to generate benchmark dataset for SkipGuide, this part might be improved to be more clear: 1. it might be more accessible to the reader if separating the shared function for matching query sequence to wild type sequence and calculating similarity into a subsection before data-A/B construction with pseudo-code; 2. In equation (1), the denominator should be the intersection between q and t to evaluate the similarity; 3. In line 292, BC_PreST is not described; 4. in line 300, is the WT target sequence T from (Pre Cas9 BC_PreUT), and what's the advantage of using 5-mers matching strategy instead of using smith waterman alignment? 5. In equation 2), are the percent spliced-in computed after the read counts normalization within sgRNA? 6. In 394 line, the VCF described both the indel and insertion type of the WT target.

Thank you for the suggestions; we’ve revised the Methods section accordingly. In response to your points:

1. We’ve added a section “Sequence Operation Notations” (section at line 395) that describes the shared functions in mathematical notations, and referred to them in the dat-A/B construction sections. Readers who seek a detailed understanding can also refer to the Jupyter notebooks provided in <https://github.com/gifford-lab/skipguide-analysis> to reproduce the described preprocessing methods.
2. The similarity metric is the Dice coefficient (<https://en.wikipedia.org/wiki/S%C3%B8rensen%E2%80%93Dice_coefficient>) operated on 5-mer sets. We’ve updated the text (line 404) to clarify this point. We’re essentially defining similarity between two sequences as the proportion of 5-mers shared between the two sequences, so the numerator and denominator should be correct.
3. Thank you very much for catching this, BC_PreST is the lookup table mapping barcodes to their set of RNA Spliced (pre Cas9) sequences. Please refer the lines 391 to 394 in the revised manuscript for the correction.
4. When we refer to WT target sequence, we’re referring to the designed sequence in lib-SA. We’ve updated the revised manuscript to clarify that Q is from BC_PostUT, and T is from lib-SA on lines 416 to 417. We also used the 5-mers similarity metric instead of smith waterman alignment for computational tractability. Consider the construction of dat-A for example. We pick a Post Cas9 Unspliced RNA transcript sequence Q, and Q is supposed to correspond to some specific designed target sequence T from lib-SA with a chunk of bases deleted due to Cas9 repair. To find out which designed T the transcript Q corresponds to, we need to compare Q to every possible T in lib-SA, and pick the one that is most similar to Q. But because there are millions of Q and thousands of designed sequences in lib-SA, it’s prohibitively expensive to perform pairwise smith waterman alignment. Instead we find that the 5-mer matching strategy is very cheap to compute and effective. We’ve clarified this point in the revised text (lines 408 to 412).
5. The percent spliced-in values are computed from dat-B datasets, which contain raw read counts of spliced transcripts with or without Exon B, and are not normalized. We only performed count normalization by total counts within gRNA groups in dat-A to obtain frequency values for each repair genotype (such that the frequencies of all repair products associated with a gRNA sums to 1).
6. Yes, the repair outcomes that we considered are 1-bp insertions at the cutsite or multiple bp deletions overlapping the cutsite. We’ve updated the VCF description at lines 518 to 522 in the revised manuscript to clarify this point.

• What's of the remaining proportion of reads count after each step of preprocessing, from regex operation, barcode with multiple sequences, and dat-A/B construction? Such a table could be provided to give a global view of lib-SA quality.

We’ve added S9 Fig to include this information, and also enhanced the sequence processing sections of the Methods to include some more details. Please refer to the entire section starting at line 356.

The read counts after each step of preprocessing is also available as code comments (and as code outputs if one tries to run them) in our released code for reproducing the results of this submission (<https://github.com/gifford-lab/skipguide-analysis>)

• Original fastq and supplementary datasets should be deposited to GEO or EBI or public lab websites.

Thank you for pointing this out. The sequencing reads have been deposited to NCBI Sequence Read Archive (SRA) under BioProject accession PRJNA647416, and will be made public upon publication. The review link is available at <https://dataview.ncbi.nlm.nih.gov/object/PRJNA647416?reviewer=7desib600hdd588j8s5kias2b1&archive=sra>

The GitHub repository links are also publicly accessible now:

<https://github.com/gifford-lab/skipguide-analysis> (contains the code and instruction for producing the results of this submission)

<https://github.com/gifford-lab/skipguide> (contains a readily usable and open source implementation of SkipGuide)

Together with the provided supplementary tables and data with this submission, we believe everything necessary for reproducibility is now available.

Reviewer #3: In this study, Louie et al. developed a novel pipeline, SkipGuide, for predicting CRISPR gRNA editing effects for exon skipping. The pipeline consists of their own inDelphi model for CRISPR editing outcome prediction and wMMSplice for exon-inclusion level prediction. Using a reporter library of 791 splice sequences and 1063 gRNAs in mESC, the authors demonstrated the high precision of SkipGuide for finding gRNAs with effectively skipped target exons.

This work addresses an essential biological problem in bridging CRISPR-Cas9 therapeutics and the splicing machinery, even though the methodological novelty is low. I would like to see it published in PLOS computational biology, pending some concerns as below.

1. As a practical proof-of-concept, it will greatly strengthen the paper’s claim if at least some endogenous exon-skipping targets’ predictions in mESC are validated.

Our experiments in this paper validated the use of gRNAs with Cas9 to ablate genome-integrated sequences (dat-A) that leads to changes in exon skipping levels (dat-B) in their native cellular context, and demonstrated SkipGuide prediction generalizability through cross validation. We have also previously validated that our high-throughput assay results match edits at endogenous loci in our work on inDelphi (Shen et al., 2018).

Therefore, we expect that CRISPR-Cas9 mediated acceptor sequence disruption to cause changes in exon skipping levels will work endogenously, but there is always a need to characterize how well it works in specific cases. In practice SkipGuide would be useful for identifying new places that can work well, and downstream high throughput experiments are necessary to characterize how well they work specifically.

We have incorporated these points in our discussion section of the revised manuscript (lines 278 to 285).

Response references:

- Shen, Max W., et al. "Predictable and precise template-free CRISPR editing of pathogenic variants." Nature 563.7733 (2018): 646-651.

2. The splicing PSI prediction is subpar especially for PSI_R. Since the goal is to predict the absolute level of PSI, using Pearson’s correlation to evaluate a predictor’s performance is insufficient. The authors should also report metrics like mean squared/absolute errors and try a few other predictors such as SpliceAI (Jaganathan et al., 2019).

Thank you for these suggestions; we’ve significantly revised the manuscript to include SpliceAI results and additional metrics such as mean squared error, mean absolute error, and root mean squared error. In the revised manuscript, we present comparisons between MaxEntScan, SpliceAI, MMSplice, and the two metamodels we’ve built, wMMSplice and MetaSplice (refer to Table 1 and Fig 3, and the results text in lines 141 to 189). As in the original manuscript, wMMSplice is a linear model that combines the predictions from the three MMSplice prediction modules. MetaSplice in the revised manuscript is just like wMMSplice, except SpliceAI predictions are also included in the linear model (refer to lines 515 to 554 in Methods). As stated in lines 44 to 49 and lines 141 to 146 of our revised manuscript, we did not test additional predictors because the recently published SpliceAI and MMSplice have demonstrated superior accuracy over existing predictors.

3. Figure 3 indicates a strong upward bias for predicted PSI_R. The authors did not address this bias nor discuss its influence on downstream analysis (which clearly persists in Figure 4). This can potentially undermine the practical utility of this work.

The region of interest for exon skipping is for small PSI_R (and small PSI_G), where MetaSplice (and SkipGuide) in our revised manuscript shows high precision. The biology and sequence determinants of exon splicing are imperfectly characterized, which leads to a bias for exon inclusion for larger PSI_R (and PSI_G). The level of precision we provide provides practically important information for gRNA selection for exon skipping, since all gRNAs selected using our model can be assayed for this property, e.g. in a similar high-throughput assay used in our study.

We’ve updated the text with some of these points. Please refer to lines 253 to 262 for our revision.

4. Figure 4 shows the high precision of predicted PSI_G, and also reveals the high False Negative rate for SkipGuide. Do these false negative predictions share any sequence and/or CRISPR editing characteristics in common?

The Fig 4 of the previous version of our manuscript shows the evaluation of SkipGuide that uses wMMSplice. In our revised manuscript, after having tried out SpliceAI and incorporating its predictions into MetaSplice, we found that SkipGuide using MetaSplice shows comparatively higher recall (due to fewer false negatives) while maintaining high precision. The Fig 4 of our previous manuscript is reproduced as S7 Fig of our revised manuscript, and the Fig 4 of our revised manuscript now shows the improved SkipGuide. There are still a large number of false negatives, but compared to the recall = 0.11 and precision = 0.91 of our previous version of SkipGuide at the 0.3 prediction threshold, the newer SkipGuide demonstrates recall = 0.20 and precision = 0.93 at the same prediction threshold.

The false negative predictions do not share any common characteristics, and they reflect the field’s incomplete understanding of the biology of splicing and limited predictive capabilities.

We’ve updated the text with some of these points. Please refer to lines 253 to 262 for our revision.

5. The authors should demonstrate that the training data used to train inDelphi (Shen et al. 2018) was not overlapped nor homologous to dataset dat-A. In particular, since inDelphi was set to run using mESC mode in this work, it’s important to show there is no training data leakage from a machine learning perspective.

Thank you for this suggestion. We performed the following analysis and confirmed that the set of sequences used to train inDelphi share very little similarity with those of dataset dat-A:

1. For every 61-bp target sequence in our dataset dat-A, find the most similar sequence in inDelphi’s training set (all 51-bp) via Smith Waterman local alignment, and compute the sequence identity between the two sequences. For local alignment, we used the same default parameters as that of BLAST’s blastn-short program (+1 match, -3 mismatch, -5 gap open, -2 gap extend). For computing sequence identity, we used the definition used by BLAST: (number of match positions in alignment) / (length of smaller sequence, 55 in this case).
2. Plot the distribution of the sequence identity values (see the newly added S2 Fig in the revised manuscript).

We found that the distribution of the best match sequence identity between dat-A and inDelphi’s training set is concentrated towards the lower end. Except for one sequence in dat-A that shares 67% sequence identity with a sequence from inDelphi’s training set, all sequences in dat-A share less than 37% sequence identity with inDelphi’s training set, with a median and mean sequence identity of 24% and 23% respectively.

We’ve added the plot of the distribution as S2 Fig in the revised manuscript, and updated the methods section (section at line 476 of the revised manuscript) with a description of this analysis.

6. It’s unclear from Figure 2B how inDelphi’s 1bp insertion model was evaluated. The panel only shows the empirical distribution for 1bp insertions.

Thank you for catching this. For predicting 1-bp insertions, inDelphi employs a nearest neighbors model that assumes genotypes of 1-bp insertions are local sequence context dependent, and are predominantly duplications of the -4 nucleotide from the NGG SpCas9 PAM. Our intention was to show in Fig 2B that this inductive bias generalizes to the 1-bp insertions observed in dat-A (which indirectly suggests that the nearest neighbor model should work quite well). However, we agree that we should also directly evaluate 1-bp insertion predictions. In the revised manuscript, we demonstrated that inDelphi’s 1-bp insertion predictions highly correlate with our observed 1-bp insertions in dat-A (r = 0.95). Please refer to lines 128 to 138, and S2 Fig of the revised manuscript.

7. Code for this work (claimed to be on GitHub) is not publicly available for evaluation.

We apologize for the oversight, but we forgot to make the GitHub repositories public. The links should be publicly accessible now:

<https://github.com/gifford-lab/skipguide-analysis> (contains the code and instruction for producing the results of this submission)

<https://github.com/gifford-lab/skipguide> (contains a readily usable and open source implementation of SkipGuide)

The sequencing reads have been deposited to NCBI Sequence Read Archive (SRA) under BioProject accession PRJNA647416, and will be made public upon publication. The review link is available at <https://dataview.ncbi.nlm.nih.gov/object/PRJNA647416?reviewer=7desib600hdd588j8s5kias2b1&archive=sra>

Together with the provided supplementary tables and data with this submission, we believe everything necessary for reproducibility is now available.

Have all data underlying the figures and results presented in the manuscript been provided?

Large-scale datasets should be made available via a public repository as described in the PLOS Computational Biology data availability policy, and numerical data that underlies graphs or summary statistics should be provided in spreadsheet form as supporting information.

Reviewer #1: None

Reviewer #2: No: The supplementary processed dataset only available to reviewer, the software is not available in public site yet. Original fastq and supplementary datasets should be deposited to GEO or EBI or public lab websites.

Reviewer #3: Yes

PLOS authors have the option to publish the peer review history of their article (what does this mean?). If published, this will include your full peer review and any attached files.

If you choose “no”, your identity will remain anonymous but your review may still be made public.

Do you want your identity to be public for this peer review? For information about this choice, including consent withdrawal, please see our Privacy Policy.

Reviewer #1: Yes: Anand K. Rampadarath

Reviewer #2: Yes: Qian Qin

Reviewer #3: No
